# Novel structures of chaos-based parallel multiple image encryption and FPGA implementation

**DOI:** 10.1038/s41598-025-30471-x

**Published:** 2025-12-14

**Authors:** Thang Manh Hoang, Pham Quang Anh, Manh-Hai Hoang, Do Quang Tran, Safwan El Assad

**Affiliations:** 1 School of Electrical and Electronic Engineering, Hanoi University of Science and Technology, 1 Dai Co Viet, 11600 Hanoi, Vietnam; 2 Department of Radiology and Diagnostic Imaging, Faculty of Medicine and Dentistry, University of Alberta, Edmonton, AB T5K 0Z4 Canada; 3 Nantes University, IETR Laboratory, Nantes, 44000 France

**Keywords:** Electrical and electronic engineering, Computer science

## Abstract

Image data has been generated massively by devices in medical imaging modalities, cameras, and even by artificial intelligence. Encryption is the powerful method to keep the image content confidential, in which an encryption algorithm must include the confusion and diffusion properties. For massive images, a efficient method of encryption must be chosen to meet the demands of encryption speed and confidentiality. So far, chaos-based image encryption has been an active topic of research because it is considered an effective method to remove the correlation in image data as well as to keep confidential by the involvement of chaotic system in the encryption process. Besides, multiple image encryption algorithms encrypt multiple images in parallel, and it provides highly efficient performance in term of speed if it is implemented on a parallel computing platform such as multiple core processing as well as digital hardware design. Chaos-based multiple image encryption is constructed by integrating a chaotic system into multiple image encryption. Recently, many algorithms of chaos-based multiple image encryption have been proposed, and they are proved to have high efficient in terms of both speed and confidentiality. However, all the existing algorithms of chaos-based multiple image encryption require images of the same size and of the same number of bits representing pixels. Further, they encrypt a cohort of plain images at the same time, and all ciphertext images of a cohort must also be decrypted at the same time. It means that if it does not allow to decrypt one or some selected ciphertext images from a cohort separately; and as a result, it wastes time and energy to decrypt unwanted images. In this paper, three novel structures of chaos-based multiple image encryption are proposed which overcome the drawbacks of existing algorithms. That is, the proposed cryptosystems accept cohort images of different sizes; pixels of images can be represented by different numbers of bits; and any selected ciphertext images from a cohort can be decrypted separately. The security is improved by using the session keys of image-content dependency. The proposed structures of multiple image encryption consist of permutation, substitution, and diffusion processes. The difference between three structures is the order of such processes. A perturbed chaotic map and a linear-feedback shift register are employed to generate pseudo-random bit sequences for session keys. The simulation results for the exemplar designs using the proposed structures show the effectiveness by means of the statistical analysis for the session keys using the NIST randomness test, information entropy, histogram, and correlation coefficients of adjacent pixels in ciphertext images, and security analysis by means of space and sensitivity of the secret key. The hardware implementation on the FPGA platform demonstrates the feasibility of the proposed structures by means of throughput and hardware efficiency.

## Introduction

Today, modern devices such as cameras, medical imaging modalities, and even artificial intelligence generate massive images. In order to keep image data confidential, encryption algorithms must be efficient in terms of speed and confidentiality in the context of massive images. So far, chaos-based image encryption is considered an effective method to remove the correlation in image data as well as to keep confidential by the involvement of chaotic system in the encryption process.

Since R. Matthews introduced the first chaos-based encryption^1^, many structures and algorithms have been proposed for chaos-based image encryption. In the early stage of development, a chaotic system was used as a pseudo-random number generator (PRNG) and chaotic values were mixed with image data to obtain ciphered images, for example^2,3^. In the next stage of development, chaotic systems were utilized in structured cryptography models, i.e., substitution-permutation networks (SPN)^4^ and Feistel networks^5^. Recently, chaos has also been used with other techniques to construct chaotic image encryption, e.g., DNA encoding^6,7^ and quantum^8,9^. However, most of the existing chaotic encryption have dealt with a single image, namely single image encryption (SIE), e.g.,^10,11^.

Rather than SIE, more efficient performance is achieved by multiple image encryption (MIE), in which multiple images, called a cohort, are encrypted simultaneously. Since 2005, MIE has been implemented in the optical domain^12–16^, and in the digital data domain, for example^17,18^. After the first chaos-based MIE was introduced^19^, many works on MIE have been reported, for example^6,20–31^. The input of chaos-based MIE algorithms is multiple images and multiple ciphertext images are achieved at the output. So far, there are several approaches to chaos-based MIE algorithms, and they are categorized based on different purposes, i.e., parallelism, techniques, image-content dependency, and structural configuration. In the following literature review, these approaches are considered only for chaos-based MIE.

- Parallelism: The purpose of designing with parallelism operations is to strive for high speed when it is implemented on parallel computing platforms such as multiple core computing or digital hardware. With respect to the level of parallelism, chaos-based MIE algorithms can be categorized into three groups depending on the processing progress of pixels from images of a cohort being encrypted. The first group of chaos-based MIE algorithms has the lowest level of parallelism, in which all original images are merged to become a large image, then the data of the big image are encrypted pixel by pixel sequentially throughout the big image. For example, the algorithm in^22,24,32–34^ is completely sequential, in which all original images are merged, and then permutation and diffusion are performed pixel by pixel through the big image. The encryption scheme in^34^ is the kind of lowest level of parallelism, in which four RGB images are merged before permutation and substitution for pixels of the big image. The second group of chaos-based MIE algorithms has the highest level of parallelism, e.g.^7^, in which pixels at the same locations from all original images are encrypted with the same progress. For example, the algorithm in^28,29^ is completely parallel if the intra-image permutation scheme is used. The third group of chaos-based MIE algorithms has a moderate level of parallelism, in which some encryption processes of algorithms can be implemented in parallel, and some others are performed sequentially, e.g.^23,31,35,36^. For example, in Karawia^23^, multiple original images with the same size and the same pixel depth are merged to become a big image; then small pieces of images that are divided from the big image are shuffled with each other; after that, small pieces of images are converted into binary vectors, which are scrambled by XORing with binary vectors obtained by chaotic values; scrambled pieces of image are merged to become the big image, before all pixels are shuffled in the range of the big image; the big image is divided into images of the same size of original images. In practice, only the step where small pieces of images are scrambled by XORing with binary vectors generated by the chaotic map can be performed in parallel, while the shuffling operations of small pieces of images and of pixels must be implemented sequentially.
- Techniques: So far, many techniques have been used in the design of chaos-based MIE. Techniques is of variety, ranged from cellular automa (CA), transforms to the deoxyribonucleic acid (DNA), and others. The technique is used with the purpose to to ensure the security, that is, to improve with the confusion and diffusion properties for chaos-based MIE. First of all, the cellular automa in^22^ is used for generating rules for the permutation process which changes the permutation key from a pixel to pixel by means of their indexes. Secondly, in the DNA technique, bits are mapped by the rules of based pairing^37^ by four DNA bases, i.e. Adenine (A), Thymine (T), Guanine (G) and Cytosine (C), and there are eight sets of encoding and decoding rules^38^. Accordingly, the computing operations based on DNA such as XOR, addition, and subtraction, are employed for the permutation and diffusion processes^6,7,22,25,39,40^. After that, the image data in the DNA bases are decoded to represent image in binary. In fact, the DNA technique is a form of binary image encryption, but it deals with groups of two bits. Thirdly, in the technique with transforms, image data is converted into transform domains and the encryption process is performed on the data in the transform domains. Then, the encrypted data is inverse transformed to obtained ciphertext images. Several transforms are used for this purpose, i.e., discrete fractional random transform^20,40,41^, Rubik cube transform^40,42^, discrete fractional cosine transform^43,44^, discrete cosine transform^32,45^, fractional discrete Fourier transform^46,47^, Krawtchouk fractional transform^48^, fractional Hartley transformation^35^, etc. However, some techniques are complicated and slow down the encryption process. For example, the transform requires a lot of computational effort, so it is not suitable for encryption with high-speed priority. The most speed is achieved when the permutation is performed in spatial domain and the diffusion process is carried out with pixel values.
- Image-content dependency: In most existing chaos-based MIE algorithms, the private key and session key are independent from the image content. Recently, there are some schemes of chaos-based MIE that are designed so the the image content is involve in the generation of the keys. There are several two main categories, i.e., secret key of image-content dependency and session keys of image-content dependency. As the first approach, the secret key of image-content dependency is the simplest way, in which some values are computed from the image, e.g. hash values, and used as the secret key. It is is kept constant during encryption. For example, the initial values and/or parameters of chaotic map are hashed values of plaintext images^26,27,33,36,40,42,49,50^. With this way, the secret key at the encryption side must be sent to the decryption side for each plaintext image. This creates the problem in the key management. In the second approach, the session keys are generated and dependent on the image content of intermediate ciphertexts during the encryption. In fact, the orbit of chaotic map is regularly perturbed by pixel values of intermediate ciphertext image while it produces the session keys as presented in^28,40,51–57^. However, most existing algorithms with the session keys of image-content dependency were designed for the chaos-based SIE. To the best our knowledge, there is only one algorithm presented in^29^ being for the chaos-based MIE. In summary, the image encryption with image-content dependency provides superior security, especially for the session keys of image-content dependency, because the encryption is sensitive to changes in the plaintext and ciphertext, in other words, it can resist from the plaintext- and ciphertext-related attacks. In addition, the session keys of image-content dependency requires more computation than the secret key of image-content dependency, so it is only suitable for the encryption algorithms that are implemented in the parallel computing platform.
- Structural configuration: So far, there are two main architectures of chaos-based MIE. The first one is based on the substitution-permutation networks (SPN) and the second one is the Feistel structure^5^. Most of algorithms of chaos-based image employs the SPN, and very few schemes use the Feistel structure^58–60^. For the SPN, the substitution provides the confusion property by a substitution box (S-box) while the diffusion property is achieved by the permutation process. It is well known that the S-box is very simple but it makes the encryption resisted from most typical attacks. Most of existing algorithms of chaos-based encryption, that are successfully analyzed, are of permutation-diffusion structure, and are without any S-box^61–65^. This means that the permutation-diffusion structure does not provide strong security enough, and an S-box should be included in the image encryption algorithm. In addition, one of the weakness in most existing chaos-based MIE algorithms is that they are designed for performing only a single round of encryption, e.g.^7,23,25,49^. It is known that the combination of diffusion and permutation has been proved efficient in saving time for chaotic iterations as presented in^33,66,67^ but, there are few chaos-based MIE algorithms designed using the combination of diffusion and permutation^29,33^. Besides, most of existing chaos-based MIE algorithms are with the structures that limit themselves to the number of grayscale images with the same size to be encrypted, i.e., two^19,20,32^, three^7,35,39,42,46,48,68^, six^40,41,44^, twelve^34^, and multiple^24,26,27,33,36,47,49,50^. In case of different sizes, the plaintext images are required to reshape or pad pixels to become the same size before being encrypted. It needs overhead time to reshape and pad additional pixels. It also wastes time and energy to encrypt, and decrypt padded pixels. In practice, there are some areas of applications such as medical imaging modalities^69^, aerial imagery^70^, etc, in which various types of images are of different sizes and different pixel depths. Even the encryption scheme presented in ^33^ allows multiple images of a cohort with any sizes, it does not accept variety of pixel depths and it encrypts images sequentially, but not in parallel.

In summary, existing chaos-based MIE algorithms still suffer from limited security, performance, and generality. For generality, most existing MIE algorithms lack generality: they rely on specific chaotic maps and particular image parameterizations, employ non-standard architectures (neither SPN nor Feistel) and perform only a single encryption round. In other words, these algorithms are designed for specific scenarios rather than as a general model.

For performance, although parallel MIE algorithms have been proposed, all encrypted images within a cohort must still be decrypted to retrieve only some of them. This implies that undesired images also need to be decrypted to access the desired ones, thereby wasting computational time and energy. Furthermore, all existing parallel MIE algorithms require plaintext images of the same size. Padding pixels with specific values is therefore necessary to make all images uniform in size, which again increases time and energy consumption during encryption and decryption. Moreover, all existing MIE algorithms assume that pixels in every image are represented with the same bit depth. However, in practice certain application domains, such as medical imaging modalities^69^ and aerial imagery^70^, involve heterogeneous images of different sizes and bit depths.

Regarding security, most existing MIE algorithms use static session keys that are independent of image content. Ideally, MIE algorithms should employ session keys that depend on the content of the image during the encryption process. This dependency makes the schemes more resistant to attacks that exploit relationships between plaintext and ciphertext, such as chosen-plaintext, known-plaintext, and chosen-ciphertext attacks. In addition, many chaotic image encryption schemes have been successfully cryptanalyzed, as reported in^61–65^. The main reason is that their diffusion processes are based on simple linear transformations between plaintext and previous ciphertext pixels. Although such linear equations provide high processing speed, they do not guarantee adequate security. Therefore, nonlinear substitution mechanisms, such as S-boxes, should be incorporated to enhance robustness.

These gaps in the existing MIE algorithms are addressed in our work. In this work, three MIE structures are proposed to overcome the disadvantages, and the main contributions and novelty are as follows.

- The proposed structures of chaos-based MIE are generic models in the form of substitution-permutation network, so that allow for employing any types of chaotic map, applying any number of encryption rounds to meet the security requirement. Three processes, i.e., permutation, diffusion, and substitution (S-box), are included in the proposed models, and those are combined in the same step and are performed pixel by pixel within individual images. The proposed models can encrypt any number of images, and work on spatial and pixel value domains. The images of a cohort are encrypted in parallel, while any ciphertext images from a cohort can be decrypted to retrieve individual images. Images can be of different sizes and different pixel depths.
- The session keys in the proposed structures are dependent on the pixel values of the intermediate ciphertext images. The chaotic map is frequently perturbed by a random number generator, and chaotic values, together with random numbers, are used to generate session keys in encryption. In addition, the substitution box (S-box) is included to enhance the confusion property of the encryption. As a result, ciphers that employ the proposed structures can withstand any type of attack that exploits the difference in the plaintexts and ciphertexts.
- The digital hardware is designed and implemented on the FPGA platform to demonstrate the effectiveness and feasibility of the proposed structures in terms of throughput and efficiency. The resultant performance is compared to other existing cryptographic hardware designs.

The remainder of the paper is organized as follows. Section Proposed MIE structures presents the details of the encryption, decryption, and operation of the proposed structures. Next, section Exemplar simulation describes the details about the cohort of images, the chaotic map, and the values of the simulation parameters. The simulation results and statistical and security analysis are discussed in this section. Then, section Digital hardware design presents details of the hardware architecture for Structure 1, including operation, and architecture of the MIE Intellectual Property and FPGA Implementation results. The throughput and efficiency of the hardware design are calculated and compared with those in other reported designs. Finally, sections Discussion and Conclusion highlight the highlights of the proposed structures in terms of operation, security, and hardware performance.

## Proposed MIE structures

Let us define $\mathbb {I}=\{I_k\mid k=1,...,K\}$ to be a set of *K* images, called a cohort, in which image $I_k=\{p(i_k,j_k)\mid i_k=1,...,M_k, j_k=1,...,N_k\}$ has a size of $M_k\times N_k$; $p(i_k,j_k)$ denotes the pixel value of image *k* at coordinates $(i_k,j_k)$, and is represented by $Q_k$ bits, called the depth of pixels. It is assumed that the images of a cohort can be of different sizes ($M_i\ne M_j$ and/or $N_i\ne N_j$ for $i\ne j$) and that the pixels of different images in a cohort have different pixel depths ($Q_i\ne Q_j$ for $i\ne j$). $I_k$ is simply considered as a matrix of values.

### Encryption

**Fig. 1:**
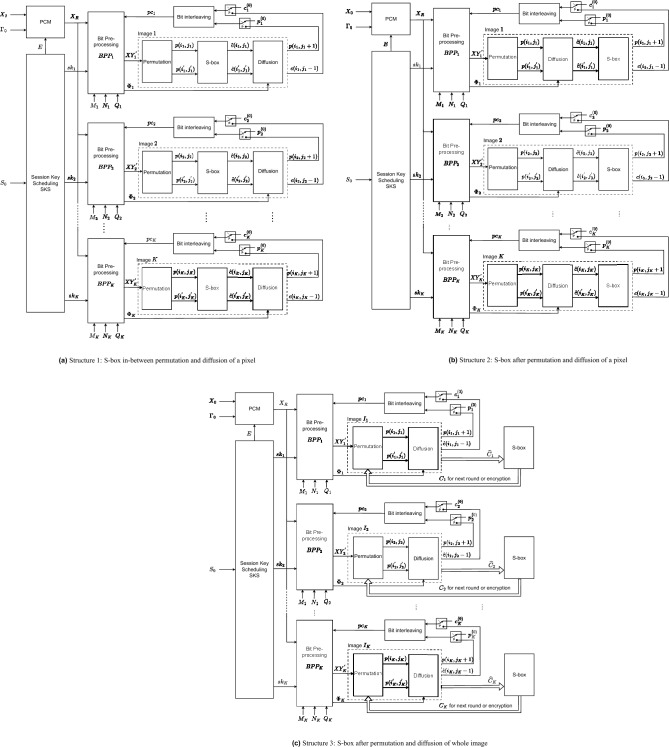
The detail of proposed structures.

As shown in Fig. 1, each of the proposed structures consists of three processes, that is, permutation, diffusion, and substitution. Here, the diffusion property is achieved by the permutation and diffusion processes, while the confusion property is obtained by the substitution process. The diffusion process depends on the session key; therefore, it also enhances the confusion property. In fact, there can be several encryption structures depending on the relative position of these processes. In this research, the permutation is fixed and performed first, and then the others to ensure that the values of all pixels are changed by the encryption. These processes are performed separately and in parallel on all images. The encryption procedure, details on processes, and the function of blocks of the proposed structures are presented in the following.

Consider encryption for *K* images at the same time. The plaintext image *k* with the size $M_k\times N_k$, $k\in \left\{ 1,2,\cdots ,K\right\}$, is denoted by $I_k=\left\{ p(i_k,j_k)|1\le i_k\le M_k, 1\le j_k\le M_k\right\}$. The source and destination locations are respectively denoted by $XY_k=(i_k,j_k)$ and $XY^{'}_k=(i^{'}_k,j^{'}_k)$, where *i* and $i^{'}$ are row numbers, and *j* and $j^{'}$ are column numbers of the image *k*, with $k\in \{1,2,\cdots ,K\}$. Intermediate-ciphertext pixels at the source and destination locations are denoted with a hat as $\widehat{c}(i_k,j_k)$ and $\widehat{c}(i^{'}_k,j^{'}_k)$, respectively. The ciphertext pixels in an encryption round at the source and destination locations are denoted by $c(i_k,j_k)$ and $c(i^{'}_k,j^{'}_k)$, respectively. The final ciphertext image *k* after $N_e$ rounds of encryption is denoted by $C_k$, which is constructed by ciphertext pixels $c(i_k,j_k)$ with $1\le i_k\le M_k$ and $1\le j_k\le M_k$.

In the encryption procedure, pixels are scanned from left to right, row by row until all pixels in an image are encrypted. All images of a cohort are encrypted separately and in parallel for $N_e$ rounds of encryption.

#### Structure 1

The first proposed structure, named Structure 1, is illustrated in Fig. 1a, in which the order of the processes is permutation, substitution, and then diffusion.

- Permutation: The permutation is to move the plain source pixel $$p(i_k,j_k)$$ and the destination pixel $$p(i^{'}_k,j^{'}_k)$$ in the same image *k*. Here, $$i_k$$ and $$j_k$$ of the source pixel are incrementally scanned from left to right and row by row of the image $$I_k$$, and $$i^{'}_k$$ and $$j^{'}_k)$$ of the destination pixel are induced from chaotic values. Details about the way in which the values of $$i^{'}_k$$ and $$j^{'}_k$$ are calculated will be shown in the next subsection.
- Substitution: The values of the permuted pixels at the source and destination locations, $p\left( i_k,j_k\right)$ and $p(i^{'}_k,j^{'}_k)$, are then changed by substitution. The substitution is carried out by the S-box to get substituted values as
  1 $$\begin{aligned} \begin{array}{ll} \widehat{c}(i_k,j_k)& =\text {S-box}(p(i_k,j_k)),\\ \widehat{c}(i^{'}_k,j^{'}_k)& =\text {S-box}(p(i^{'}_k,j^{'}_k)), \end{array} \end{aligned}$$
  where $\text {S-box(.)}$ is the function that represents the substitution process and returns the substitution values.
- Diffusion: After being replaced by Eq. (1), the pixels at the source and destination locations, $\widehat{c}(i_k,j_k)$ and $\widehat{c}(i^{'}_k,j^{'}_k)$, are diffused to obtain ciphertext pixels as
  2 $$\begin{aligned} \begin{array}{ll} c(i_k,j_k)& =F^{(Diff)}_k\left( \widehat{c}(i_k,j_k),c(i_k,j_k-1),\phi ^{(S)}_k\right) ,\\ c(i^{'}_k,j^{'}_k)& =F^{(Diff)}_k\left( \widehat{c}(i^{'}_k,j^{'}_k),c(i^{'}_k,j^{'}_k-1),\phi ^{(D)}_k\right) , \end{array} \end{aligned}$$
  where $F^{(Diff)}_k(.)$ is the diffusion function that operates bit-wise; $c(i_k,j_k-1)$ and $c(i^{'}_k,j^{'}_k-1)$ are the values of the ciphertext pixels that stand immediately before the source and destination locations, respectively; and $\phi ^{(S)}_k$ and $\phi ^{(D)}_k$ are pseudo-random values for the source and destination pixels. Note that the superscripts (*S*) and (*D*) are denoted for the source and destination in the following text.

In Structure 1, every source pixel is subsequently performed by three processes as described above. It is clear that the processes of permutation, substitution, and diffusion are combined and performed pixel-by-pixel. All images of a cohort are encrypted separately and in parallel for $N_e$ encryption rounds.

#### Structure 2

The second proposed structure, named Structure 2, is presented in Fig. 1b. After permuting pixels, the source and destination pixels are diffused and then substituted as specified below.

- Permutation: The permutation of Structure 2 is the same as that in Structure 1.
- Diffusion: Pixels at the source and destination locations $p(i_k,j_k)$ and $p(i^{'}_k,j^{'}_k)$ are diffused by
  3 $$\begin{aligned} \begin{array}{ll} \widehat{c}(i_k,j_k)& =F^{(Diff)}_k\left( p\left( i_k,j_k\right) ,c\left( i_k,j_k-1\right) ,\phi ^{(S)}_k\right) ,\\ \widehat{c}(i^{'}_k,j^{'}_k)& =F^{(Diff)}_k\left( p(i^{'}_k,j^{'}_k),c(i^{'}_k,j^{'}_k-1),\phi ^{(D)}_k\right) , \end{array} \end{aligned}$$
  where function $F^{(Diff)}_k(.)$, pixels $c(i_k,j_k-1)$, $c(i^{'}_k,j^{'}_k-1)$, and chaotic values $\phi ^{(S)}_k$ and $\phi ^{(D)}_k$ are defined in Eq. (2).
- Substitution: The diffused pixels at the source and destination locations, $\widehat{c}(i_k,j_k)$ and $\widehat{c}(i^{'}_k,j^{'}_k)$, are then changed by substitution. The substitution is carried out by the S-box to get ciphertext pixels as
  4 $$\begin{aligned} \begin{array}{ll} c(i_k,j_k)& =\text {S-box}(\widehat{c}(i_k,j_k)),\\ c(i^{'}_k,j^{'}_k)& =\text {S-box}(\widehat{c}(i^{'}_k,j^{'}_k)). \end{array} \end{aligned}$$

Compared with Structure 1, the diffusion process of Structure 2 is carried out before substitution. The encryption procedure is also performed pixel by pixel.

#### Structure 3

The third proposed structure, named Structure 3, is illustrated in Fig. 1c, in which permutation and diffusion are combined and performed pixel by pixel, the same as those in Structure 2. After all pixels of an image are permuted and diffused, a substitution is then performed on all pixels separated from permutation and diffusion. Specifically, the encryption processes are as follows.

- Permutation: The permutation of Structure 3 is the same as that in Structure 1.
- Diffusion: The permutation of Structure 3 is the same as that in Structure 2 as given in Eq. (3). After all pixels of the image are permuted and diffused, the intermediate image $\widehat{C}_k=\left\{ \widehat{c}(i_k,j_k)|1\le i_k\le N_k, 1\le j_k\le M_k\right\}$ is obtained.
- Substitution: The pixels in the intermediate image $\widehat{C}_k$ are substituted by the S-box as
  5 $$\begin{aligned} \begin{array}{ll} c(i_k,j_k)&=\text {S-box}\left( \widehat{c}(i_k,j_k)\right) . \end{array} \end{aligned}$$
  where $\widehat{c}(i_k,j_k)$ is the pixel in location $XY_k=(i_k,j_k)$ of $\widehat{C}_k$.

#### Remarks

The highlight of the proposed structures can be derived from the configuration as follows.

- More than one process is combined and performed at the same time. Those processes are permutation, substitution, and diffusion in Structure 1; permutation, diffusion, and substitution in Structure 2; and permutation and diffusion in Structure 3. The combination of more than one process is the difference in the proposed structures from most existing encryption algorithms. As a result, chaotic values are efficiently used for the processes of permutation and diffusion, and the speed is improved compared to that of most existing encryption algorithms in which all pixels of an image are completed for a process before the next process is performed, where it requires chaotic iterations for separate processes.
- As illustrated in Fig. 1, the proposed structures encrypt images separately and in parallel. Therefore, the various sizes of the images can be accepted. In addition, because the session keys are generated separately for multiple images, the decryption can be done separately for any selected images. In addition, encryption separation allows separation of the decryption, which is the unique feature of the proposed structures.

### Decryption

The decryption structures have the same block functions except that the order of processes is reversed compared to that in the encryption. Specifically, the permutation is the same as in the encryption while inverse substitution and inverse diffusion are used. In addition, in every individual image, the direction of the pixel scanning must be reversed in compared with that in the encryption. The decryption is also performed in $N_e$ rounds.

All blocks of the proposed structures that provide parameters for the encryption processes are described in detail below.

### Functions of blocks in the proposed structures

A chaotic map is used to generate pseudo-random values, and its dynamics of a chaotic map is perturbed by bits from the block Session Key Scheduling (SKS), the so-called perturbed chaotic map (PCM). The PCM and SKS are initiated and then iterated to generate bit sequences for permutation and diffusion during encryption. As shown in Fig. 1, Bit Pre-processing of image *k*, $BPP_k$, uses bits of next plaintext, $p(i_k,j_k+1)$, previous ciphered pixels, $c(i_k,j_k-1)$, and the SKS bit sequence, $sk_k$, to generate the coordinates of the destination pixel for permutation ($XY^{'}_k$) and pseudo-random values ($\Phi _k$) for diffusion to encrypt the present pixel of image *K*. The functions of blocks in the proposed structures are explained as follows.

#### Perturbed chaotic map (PCM)

The general model of PCM is employed in the proposed structures expressed by

6 $$\begin{aligned} \left\{ \begin{array}{ll} X_{n+1}& =F(X_n,\widehat{\Gamma }_n),\\ \widehat{\Gamma }_n& =\Gamma _0 \oplus \Delta _{\Gamma _n},\\ \Delta _{\Gamma _n}& =\left\{ \begin{array}{ll} Y_1(E) & \text {for } n=1,\\ Y_2(X_{n}) & \text {for } n> 1, \end{array} \right. \end{array} \right. \end{aligned}$$

where *F*(.) is a chaotic function; $Y_i$ with $i=1,..,4$ are bit arrangements in the construction of bit sequences of perturbation; $X_n=[x_n^{(D)}$ $x_n^{(D-1)}...$ $x_n^{(2)}$ $x_n^{(1)}]^T$ is the vector of state variables; $\Gamma _0=[\gamma _0^{(G)}$ $\gamma _0^{(G-1)}...$ $\gamma _0^{(2)}$ $\gamma _0^{(1)}]^T$ is the vector of initial control parameters and is regularly perturbed by $\Delta _{\Gamma _n}=[\delta _{\gamma ^{(G)}_n}$ $\delta _{\gamma ^{(G-1)}_n}...$ $\delta _{\gamma ^{(2)}_n}$ $\delta _{\gamma ^{(1)}_n}]^T$. *D* and *G* are the numbers of dimensions and those of the control parameters of the chaotic map, respectively. $\widehat{\Gamma }_n=[\widehat{\gamma }_n^{(G)}$ $\widehat{\gamma }_n^{(G-1)}...$ $\widehat{\gamma }_n^{(2)}$ $\widehat{\gamma }_n^{(1)}]^T$ is the vector of perturbed control parameters^53^.

**Fig. 2:**
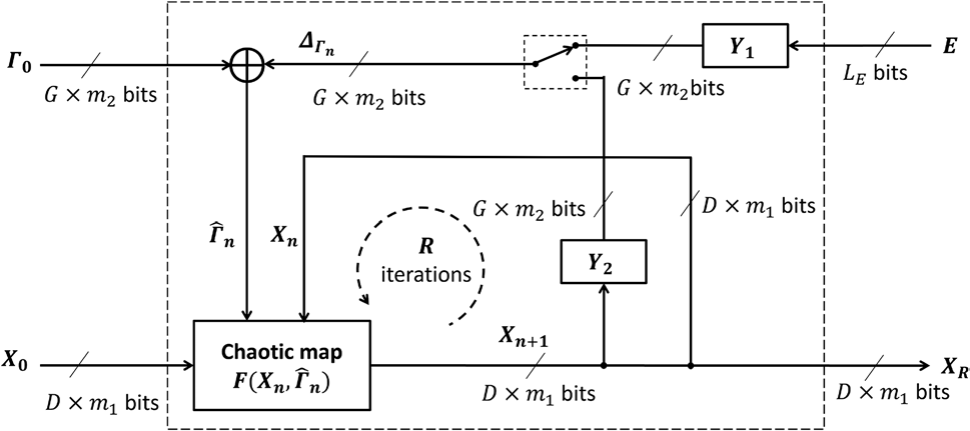
The configuration of the PCM^53^.

Figure 2 illustrates the general PCM configuration, in which $Y_i$, $i=1,2$, performs the bit arrangement so that the bit of $X_n$ is extracted and used to perturb the chaotic map. The bit sequence *E* with $L_E=L_X+L_{\Gamma }$ bits in length is used as an external perturbation. The values of $x_n^{(d)}$ and $\gamma _n^{(g)}$, with $d=1,...,D$ and $g=1,...,G$, are represented by the $m_1$ and $m_2$ bits, respectively. The values of $x_n^{(d)}$ and $\gamma _n^{(g)}$ are real, so the numbers of bits for the integer and fraction portions of $x_n^{(d)}$ and $\gamma _n^{(g)}$ are denoted by $\left\langle m^{(int)}_1,m^{(frac)}_1\right\rangle$ and $\left\langle m^{(int)}_2,m^{(frac)}_2\right\rangle$, respectively, with $m_1=m^{(int)}_1+m^{(frac)}_1$ and $m_2=m^{(int)}_2+m^{(frac)}_2$ as

7 $$\begin{aligned} \begin{array}{ll} x_n^{(d)}& =\sum ^{m^{(int)}_1-1}_{i=-m^{(frac)}_1} a_i.2^i,\\ \gamma _n^{(g)}& =\sum ^{m^{(int)}_2-1}_{i=-m^{(frac)}_2} b_i.2^i. \end{array} \end{aligned}$$

where $a_i$ and $b_i$ are binary. The amounts of perturbation, $\delta _{x_n^{(d)}}$ and $\delta _{\gamma _n^{(g)}}$, are

8 $$\begin{aligned} \begin{array}{ll} \delta _{x_n^{(d)}}& =\sum ^{m^{(int)}_1-1}_{i=-m^{(frac)}_1} c_i.2^i,\\ \delta _{\gamma _n^{(g)}}& =\sum ^{m^{(int)}_2-1}_{i=-m^{(frac)}_2} d_i.2^i. \end{array} \end{aligned}$$

where $c_i$ and $d_i$ are binary. Similarly, the values of $\widehat{X}_n$ and $\widehat{\Gamma }_n$ are represented by

9 $$\begin{aligned} \begin{array}{ll} \widehat{x}_n^{(d)}=\sum ^{m^{(int)}_1-1}_{i=-m^{(frac)}_1} e_i.2^i & \text { for } j=1,...,D\\ \widehat{\gamma }_n^{(g)}=\sum ^{m^{(int)}_2-1}_{i=-m^{(frac)}_2} f_i.2^i & \text { for } k=1,...,G. \end{array} \end{aligned}$$

where $e_i$ and $f_i$ are also binary. The perturbation is the XORing operation as follows.

10 $$\begin{aligned} \begin{array}{ll} e_i=a_i\oplus c_i & \text { for } i=-m^{(frac)}_1,...,m^{(int)}_1-1,\\ f_i=b_i\oplus d_i & \text { for } i=-m^{(frac)}_2,...,m^{(int)}_2-1. \end{array} \end{aligned}$$

The value ranges of state variables and control parameters must be constrained in those as in the original model. So, a certain number of bits in the values of $x_n^{(d)}$ and $\gamma _n^{(g)}$ are fixed with specified values to ensure that the PCM exhibits chaotic behavior, while the other bits can be flippable. The positions of the perturbed bits are specified by $Y_i$.

In this work, PCM is initiated by $X_0$ and $\Gamma _0$, and iterates for *R* times to get $X_R$ to encrypt a set of *K* pixels; each pixel comes from an image. This process is repeated until all pixels in every image are encrypted for $N_e$ rounds of encryption. More details on PCM can be found in^53^.

#### Session key scheduling (SKS)

s The SKS produces session keys to encrypt individual pixels and perturb the PCM. The core of SKS is the keystream generator based on the linear feedback shift register (LFSR)^71^.

The input of the keystream generator is the initial vector $S_0$ and the predefined private key *ID*. The SKS outputs are the session keys $sk_k$ with $k=1,...,K$ and the bit sequence *E* for PCM perturbation. The bit sequence $sk_k$ will be used to identify the location of destination pixels ($XY^{'}_k$) and to generate diffusion values ($\Phi _k$), so the length of $sk_k$ must be

11 $$\begin{aligned} L_{sk}=\lceil \textrm{log}_2(N_{pix\_max})\rceil +2Q_{max}~~\text {(bits)} \end{aligned}$$

where $N_{pix\_max}=M_{max}*N_{max}$ is with $M_{max}=\textrm{max}_{k\in \{1,2,...,K\}}M_k$ and $N_{max}=\textrm{max}_{k\in \{1,2,...,K\}}N_k$; $Q_{max}$ is the maximum number of bits that represent a pixel of images, $Q_{max}=\textrm{max}_{k\in \{1,2,...,K\}}Q_k$; and $\lceil .\rceil$ is the round-up function.

The length $L_E$ requires $\left( K*L_{sk}+L_E\right)$ bits to encrypt a group of *K* pixels from *K* images at the same time. Therefore, the sequence requires at least $(K*L_{sk}+L_E)*N_{pix\_max}$ bits to encrypt all pixels of *K* images. Consequently, the number of states of LFSR^72^ must be at least

12 $$\begin{aligned} N_{LFSR}=\lceil \textrm{log}_2\left( (K*L_{sk}+L_E)*N_{pix\_max}\right) \rceil ~~\text {(states)}. \end{aligned}$$

**Fig. 3:**
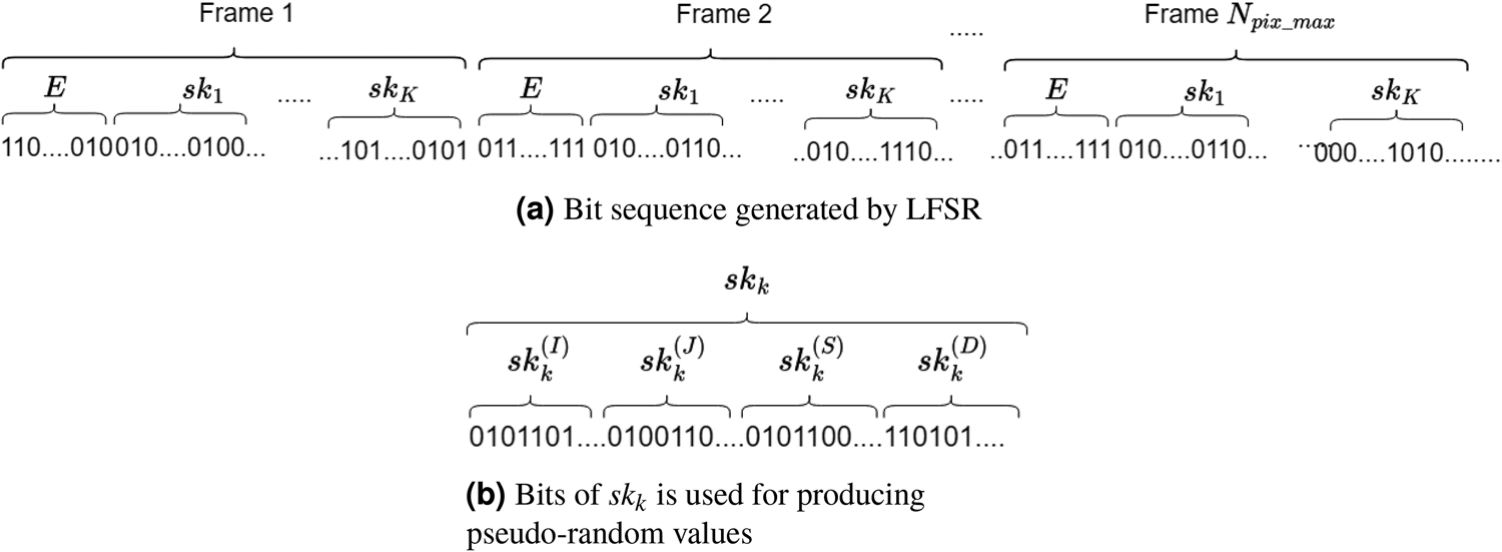
Arrangement of the keystream in the SKS.

As shown in Fig. 3a, each frame is of $K*L_{sk}+L_E$ bits to encrypt *K* pixels from *K* images. So, it requires $N_{pix\_max}$ frames to encrypt all pixels of *K* images. In detail, each sequence $sk_k$ is used to produce pseudo-random values, as shown in Fig. 3b.

The bit sequence *E* is used to construct $\delta _{x^{(d)}_n}$ and $\delta _{\gamma ^{(g)}_n}$ to perturb the PCM as shown in Eq. (8). The length of *E*, $L_E$, is considered using the numbers of bits represented for the values of the state variables, and the control parameters are $D*m_1$ and $G*m_2$, respectively. However, some constraints are made for the range of state variables and control parameters of the PCM to ensure the existence of chaotic behavior. In terms of security, the number of bits that are extracted to construct $XY^{'}_k$ and $\Phi _k$ should be lower than $D*m_1$, so the attacker is challenged to recover chaotic dynamics and infer values of $X_0$ and $\Gamma _0$.

#### Bit Pre-processing (BPP)

The BPP takes bits from $X_R$, $sk_k$, and $pc_k$ to produce the coordinate of the destination pixel, $XY^{'}_k=(i^{'}_k,j^{'}_k)$, for permutation with the pixel at $(i_k,j_k)$ of the image *k* as

13 $$\begin{aligned} \begin{array}{ll} i_k^{'}& =mod(F^{(IJ)}_k(bs^{(I)}_k,sk^{(I)}_k,pc_k),M_k)+1,\\ j_k^{'}& =mod(F^{(IJ)}_k(bs^{(J)}_k,sk^{(J)}_k,pc_k),N_k)+1, \end{array} \end{aligned}$$

and pseudo-random values, $\Phi _k=\{\phi ^{(S)}_k,\phi ^{(D)}_k\}$, for diffusion of source and destination pixels as

14 $$\begin{aligned} \begin{array}{ll} \phi ^{(S)}_k& =F^{(\Phi )}_k(bs^{(S)}_k,sk^{(S)}_k,pc_k),\\ \phi ^{(D)}_k& =F^{(\Phi )}_k(bs^{(D)}_k,sk^{(D)}_k,pc_k), \end{array} \end{aligned}$$

where $F^{(IJ)}_{k}(.)$ and $F^{(\Phi )}_k(.)$ are bitwise functions of image *k*. bit sequences $bs^{(I)}_k$, $bs^{(J)}_k$, $bs^{(S)}_k$, and $bs^{(D)}_k$ are extracted from the values of $X_R$; bit sequence $pc_k$ is formed by interleaving bits of pixels $p(i_k,j_k+1)$ and $c(i_k,j_k-1)$ as given in Eq. (16); $M_k$ and $N_k$ are the actual size of the image $I_k$, and each pixel is represented by $Q_k$ bits; and *mod*(.) is the modulus function.

#### Bit interleaving

The block *Bit interleaving* is used to interleave bits of the next plain pixel $p(i_k,j_k+1)$ and the past ciphertext pixel $c(i_k,j_k-1)$ of the image $I_k$, and a bit sequence $pc_k$ is obtained. Specifically, the representation of $p(i_k,j_k+1)$ and $c(i_k,j_k-1)$ is

15 $$\begin{aligned} \begin{array}{ll} p(i_k,j_k+1)& =\sum ^{q_k-1}_{i=0} b^{(p)}_i.2^i,\\ c(i_k,j_k-1)& =\sum ^{q_k-1}_{i=0} b^{(c)}_i.2^i,\\ \end{array} \end{aligned}$$

where $b^{(p)}_i$ and $b^{(c)}_i$ are binary. The bitwise operation of interleaving is as follows.

16 $$\begin{aligned} \begin{array}{ll} pc_k=b^{(p)}_{q_k-1}b^{(c)}_{q_k-1}b^{(p)}_{q_k-2}b^{(c)}_{q_k-2}...b^{(p)}_{0}b^{(c)}_{0}=||^{q_k-1}_{i=0}b^{(p)}_{i}b^{(c)}_{i},\\ \end{array} \end{aligned}$$

where || is the bit concatenation operation. The initial ciphered pixels $c^{(0)}_k$ and the plain pixels $p(M_k,N_k+1)$ denoted by $p^{(0)}_k$ for $\forall k\in \{1,2,...,K\}$ are used to encrypt the pixels at (1, 1) and the last pixel at $(M_k,N_k)$, respectively. Those are part of the secret key.

**Fig. 4:**
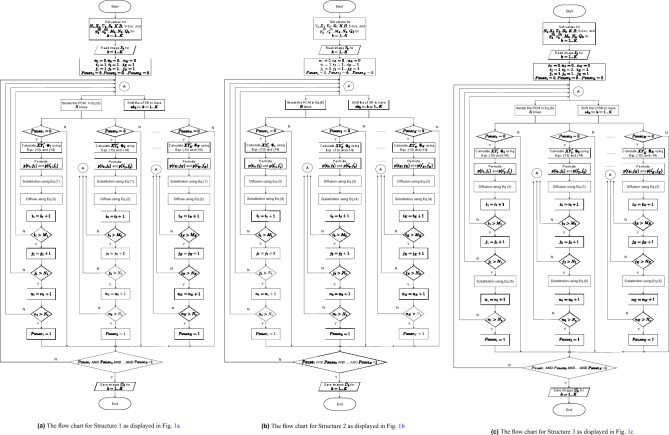
The flowchart of the encryption.

### Operation and security of the proposed structures

In this subsection, the operation of the structures proposed in Fig. 1 is described step by step in the form of a flow chart as in Fig. 4. The security in terms of the secret key space and the image-content dependency is discussed.

#### The operation

The flow charts in Fig. 4 show that the encryption is separated for each image and in parallel for multiple images. The inputs are the parameters’ values, including the number of encryption rounds ($N_e$), the initial vectors and control parameters of the chaotic map ($X_0$ and $\Gamma _0$), the number of cohort images (*K*) and the size of images ($M_k$, $N_k$ and $Q_k$), the number of chaotic iterations (*R*), the substitution box (S-box), initial secret values ($p^{(0)}_k$, $c^{(0)}_k$). From that, running values are set for the encryption of *K* images.

Each encryption session is separate for an individual image. For each image encryption session, $Pause_k$ is the flag that indicates that the encryption process for image $I_k$ is in progress ($Pause_k=0$) or complete ($Pause_k=1$). In an encryption progress for the image $I_k$, the processes of permutation, diffusion, and substitution are performed in the orders described in Subsection Description for the encryption.

As shown in the flowchart for Structures 1 and 2 in Fig. 4a and 4b, pixels are chosen to encrypt sequentially, that is, scanning from left to right and row by row. Each pair of pixels, source and destination pixels, in Structures 1 and 2 is processed by combining the permutation, diffusion, and substitution in one step. Then, the next pair of pixels is processed as indicated by the increment of $i_k$ and $j_k$. During each process, appropriate equations as described for Structures 1 and 2 in Subsection Description for the encryption are used. If all pixels of $I_k$ are scanned and encrypted for rounds $N_e$, that is, $n_k>N_e$, the encryption of the image $I_k$ is complete by setting the flag $Pause_k=1$.

The flowchart in Fig. 4c for Structure 3 shows that the permutation and diffusion processes are carried out the same as those in Structures 1 and 2, except that the substitution process is separated from the permutation and diffusion. The substitution process is performed after all pixels of $I_k$ are permuted and diffused.

The encryption for all images is finished if all the flags are set to ‘1’, that is, $Pause_k=1$ for $\forall k\in \{1,2,\cdots ,K\}$. Specifically, *K* encryption sessions are performed in parallel for *K* images. The chaotic map is iterated while the LFSR is shifted at the same time. The session keys are generated in parallel for *K* encryption sessions.

It is clear from the structural configuration and flow charts of the encryption algorithms that all images are encrypted separately and in parallel. Therefore, three features are achieved; that is, it allows images with different sizes and different pixel depths, and it allows decrypting some ciphertext images of a cohort, instead of decryption for all ciphertext images as in existing algorithms. In addition, these structures can be easily implemented on the parallel computing platform, such as multiple cores and hardware design, to achieve high-speed encryption.

#### The security

Consider the security of the proposed structures in terms of secret key space and image-content dependency. Firstly, the secret key space of the proposed structures is considered by several factors, i.e. the initial vectors of state variables and control parameters of the chaotic map, the LFSR’s seed, initial values of ciphertexts $c^{(0)}_k$ and plaintexts $p^{(0)}_k$ with $k=1,2,\cdots ,K$ as in Eq. (15), and the user key of the block *SKS*. Specifically, the number of bits for the LFSR’s seed and the user key can be chosen as desired, but it must satisfy the number of states given in Eq. (12) to ensure randomness of session keys. The initial values of $c^{(0)}_k$ and $p^{(0)}_k$ with $k=1,2,\cdots ,K$ have a fixed number of bits. For example, 64 bits for $c^{(0)}_k$ and 64 bits for $p^{(0)}_k$ with $K=8$ and 8-bit grayscale pixels. The initial vectors of the chaotic map consist of the initial value of the state variables, $X_0$, and the initial value of the control parameters, $\Gamma _0$. Here, the chaotic map is implemented on a digital platform, so $X_0$ and $\Gamma _0$ are represented by a number of bits. The chaotic map is perturbed as shown in Fig. 2, therefore, the bits that are changed during the iteration of the chaotic map are considered the secret key. The number of bits of the secret key can be as long as desired and depending on the representation for the values of state variables and control parameters. In addition, a chosen chaotic map whose state variables and control parameters have a large value range of chaotic behavior also provides a large number of bits for the secret key. Moreover, depending on the chaotic map adopted, higher dimensions of state space can provide a large number of bits for the secret key. For example, the Logistic map has only one state variable, and the value range of chaotic variable is in the range 0 to 1. Therefore, the number of bits for the secret key is dependent on the number of bits representing the values of the state variable and control parameters. In contrast, the Cat map and the Standard map are two-dimensional state variables, and the value ranges of state variables and control parameters that exhibit chaos are larger than the Logistic map. Therefore, the Cat map and Standard maps can provide a number of bits for the secret key larger than that from the Logistic map. In summary, the number of bits for the secret key or the key space can be as large as desired in several ways, as given above. However, adoptions of a large number of bits representing values of state variables and control parameters and/or higher-dimensional chaos pay the speed of encryption. It takes longer to compute for an iteration of the chaotic map.

Secondly, the proposed MIE algorithms have the image-content dependency. Specifically, the current session keys are the coordinates of destination pixels $XY^{'}_k$ and pseudo-random values $\Phi _k$ with $k=1,2,\cdots ,K$. For image $I_k$, $XY^{'}_k$ and $\Phi _k$ in Eqs. (13) and  (14) are directly related to the values of the previous ciphertext pixel $c(i_k,j_k-1)$ and the next plaintext pixel $p(i_k,j_k+1)$ by means of $pc_k$ as given in Eq. (16). This means that the permutation and diffusion processes depend on the image content. The dependency on image content provides better security and resistance to the types of attacks such as chosen-plaintext and chosen-ciphertext attacks. However, the algorithms of proposed structures continually encrypt pixel-by-pixel throughout images, so they cannot resist the ciphertext-crop attack.

## Exemplar simulation

In this section, examples of all proposed structures are simulated using Python v3.12.6, and then statistical and security analyzes are performed using Matlab R2016a for the simulation results to confirm effectiveness.

### Chosen chaotic maps, images and values of parameters for simulation

**Fig. 5:**
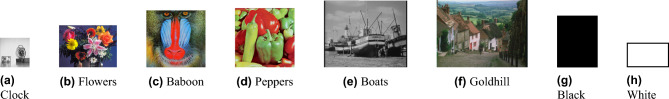
Exemplar set of images with actual ratios.

In this example, a set of eight images as shown in Fig. 5 is encrypted using each of the three proposed designs. Table 1 shows the size of the image and the number of bits that represent pixels in different images. As given in Table 2, the critical values for encryption are to specify its capability to adopt the values of other parameters. $K_{max}$, $M_{max}$, $N_{max}$, and $Q_{max}$ are, respectively, the maximum numbers of images, rows, and columns of pixels, and of bits representing a pixel. Here, the chosen values are greater than or at least equal to the actual numbers characterized by the images.

**Table 1:** Parameters of chosen images.

Image	Color	Size ($M_k\times N_k$)	#bits per pixel ($Q_k$)
Clock	GrayScale	256$\times$256	8
Flowers	RGB	362$\times$500	24
Baboon	RGB	480$\times$500	24
Peppers	RGB	512$\times$512	24
Boats	GrayScale	576$\times$720	8
Goldhill	RGB	532$\times$720	24
Black	GrayScale	790$\times$616	8
White	GrayScale	492$\times$874	8

**Table 2:** Critical values for the exemplar encryption.

Highest parameter	Value	Unit
$K_{max}$	16	images
$M_{max}$	1024	rows
$N_{max}$	1024	columns
$N_{pix\_max}$	$2^{10}\times 2^{10}$	pixels
$Q_{max}$	24	bits

- Chosen chaotic maps: The Cat map is chosen and its perturbation is described in the form of Eq. (6) as
  17 $$\begin{aligned} \left\{ \begin{array}{ll} \left[ \begin{array}{c} x^{(2)}_{n+1}\\ x^{(1)}_{n+1}\end{array} \right] & = mod\left( \left[ \begin{array}{c} \widehat{\gamma }_n^{(1)}x^{(2)}_n+\left( 1+\widehat{\gamma }_n^{(2)}\widehat{\gamma }_n^{(1)}\right) x^{(1)}_n\\ x^{(2)}_n + \widehat{\gamma }_n^{(2)}x^{(1)}_n\end{array}\right] ,1\right) ,\\ \left[ \begin{array}{c} \widehat{\gamma }^{(2)}_{n}\\ \widehat{\gamma }^{(1)}_{n} \end{array} \right] & = \left[ \begin{array}{c} \gamma ^{(2)}_{0}\\ \gamma ^{(1)}_{0} \end{array} \right] \bigoplus \left[ \begin{array}{c}\delta _{\gamma ^{(2)}_{n}}\\ \delta _{\gamma ^{(1)}_n} \end{array} \right] , \\ \left[ \begin{array}{c}\delta _{\gamma ^{(2)}_{n}}\\ \delta _{\gamma ^{(1)}_{n}} \end{array} \right] & = \left\{ \begin{array}{ll} \left[ \begin{array}{c} Y^{(a)}_1\left( E\right) \\ Y^{(b)}_1\left( E\right) \end{array} \right] & \text {for } n=1, \\ \\ \left[ \begin{array}{c} Y^{(a)}_2\left( x^{(2)}_{n}\right) \\ Y^{(b)}_2\left( x^{(1)}_{n}\right) \end{array} \right]&\text {for } 1< n \le R, \end{array} \right. \end{array} \right. \end{aligned}$$
  where $X_n=\left[ x^{(2)}_{n}~~x^{(1)}_{n}\right] ^\intercal$ are state variables and $\Delta _{\Gamma _n}=\left[ \gamma ^{(2)}_{n}~~\gamma ^{(1)}_{n}\right] ^\intercal$ are control parameters; these are in the ranges of $x^{(2)}_{n}$, $x^{(1)}_{n} \in (0,1)$ and $\gamma ^{(2)}_{n}$, $\gamma ^{(1)}_{n}>0$; $Y^{(a)}_2$, $Y^{(b)}_2$, $Y^{(a)}_1$ and $Y^{(b)}_1$ are to arrange bits of either *E* or $X_n$ to construct permutation amounts. The values of the state variables and control parameters are represented as fixed-point numbers. The formats, bit patterns, value ranges, and bit positions to be perturbed are given in Table 3. $Y^{(a)}_1$ and $Y^{(b)}_1$ are chosen the same format as $Y^{(a)}_2$ and $Y^{(b)}_2$, respectively, but bits are from *E*. Bit arrangements $Y^{(a)}_2$ and $Y^{(b)}_2$ are chosen to construct perturbation amounts $\Delta _{\Gamma _n}$ as shown in Table 4; where $B_0$ at specific positions are fixed at ‘0’, and the group $(i,b_j)$ is denoted for bit $b_j$ of the state variable $x^{(i)}_n$. These are bits used as $d_i$ in Eq.(8), and the perturbation is based on the XOR operation as given in Eq. (10). The initial values in decimal for the PCM are chosen as in Table 5. It is noted that even though the value range of control parameters are separated and the perturbation is applied, the chaotic behavior certainly exists with nonstationary dynamics.
- The SKS: From Table 2, the maximum number of pixels in each image is $N_{pix\_max}=2^{20}$ or 2048 pixels. According to Eq. (11), the length of $sk_k$ is $L_{sk}=68$ bits. As assumed that each of the state variables and control parameters of the PCM is perturbed by 16 bits as given in the leftmost column of Table 3, therefore, the length of bit sequences to perturb the PCM is $L_E=64$ bits for the perturbed Cat map. In this example, the LFSR is chosen and, according to Eq. (12), the number of states of LFSR is $N=32$ with the primitive polynomial $X^{32}+X^{22}+X^{2}+1$ as suggested in ^72^. The seed of LFSR is $S_0$ in hexadecimal form, as shown in Table 5.
- The BPP: Table 6 shows bits of chaotic values, $b_{-17}$...$b_{-48}$, being extracted and used to construct values of coordinates of destination pixels and pseudo-random values in Eqs. (13) and (14), respectively. More specifically, bits sequences $bs^{(t)}_k$ with $t\in \{I,J,S,D\}$ in the four leftmost columns of Table 6 are taken bits of chaotic values in the second column. Then, bit sequences $bs^{(t)}_k$ with $t\in \{I,J,S,D\}$ are XORed with portions of $sk_k$ (see Fig. 3b) to produce pseudo-random values for permutation and diffusion. The modulus is applied as given in Eqs. (13) and (14) for smaller images and smaller number of bits representing the pixels. The XOR operation is chosen for functions $F_k^{(IJ)}$ and $F_k^{(\Phi )}$ in Eqs. (13) and (14) as $F_k^{(IJ)}=bs_k^{(t_1)}\oplus sk_k^{(t_1)} \bigoplus _{i=1}^T pc^{(i)}_k$ and $F_k^{(\Phi )}=bs_k^{(t_2)}\oplus sk_k^{(t_2)} \bigoplus _{i=1}^T pc^{(i)}_k$, with $t_1\in \{I,J\}$ and $t_2\in \{S,D\}$. Here, the bit sequences $sk_k^{(t_1)}$ and $sk_k^{(t_2)}$ can be cut to shorten and fit the lengths of $bs_k^{(t_1)}$ and $bs_k^{(t_2)}$, respectively. Also, bit sequence $pc_k$ is padded with bits 1’s to have the length equal to an integer number of times of the length of $bs_k^{(t)}$ with $t\in \{I,J,S,D\}$, then it is divided into *T* sub-sequences before XORing all together.
- The diffusion: Similarly to the functions in the BPP, the function $F^{(Diff)}_k(.)$ is the XOR operation among its entries in Eqs. (2) and (3) as $F_k^{(Diff)}=\widehat{c}(i_k,j_k)\oplus c(i_k,j_k-1) \oplus \phi _k^{(t)}$ and $F_k^{(Diff)}=p(i_k,j_k)\oplus c(i_k,j_k-1)\oplus \phi _k^{(t)}$, respectively, with $t\in \{S,D\}$.
- The S-box: In this exemplar simulation, the 8-bit AES S-box^73^ and its corresponding inverse S-box are chosen. In fact, a new S-box can be generated by using chaotic values. For pixels represented by 24-bit data, i.e. red, green, and blue colors, three consecutive substitutions using the same S-box are performed, such that each is for 8 bit data of pixels. Then, the outputs of the S-box are merged to return 24-bit data of pixels.

**Table 3:** Representation of state variables and control parameters, their value ranges, and initially perturbed bits.

Parameter	Format	Pattern in binary	Value range	Position of 16 perturbed bits
$\gamma _n^{(2)}$	$\left\langle 52,49\right\rangle$	xx1(.)xxx...xx	[1.0,2.0), [3.0,4.0), [5.0,6.0), and [7.0,8.0)	$b_{2}b_{1}b_{-1}...b_{-13}b_{-14}$
$\gamma _n^{(1)}$	$\left\langle 52,49\right\rangle$	xx1(.)xxx...xx	[1.0,2.0), [3.0,4.0), [5.0,6.0), and [7.0,8.0)	$b_{2}b_{1}b_{-1}...b_{-13}b_{-14}$
$x_n^{(2)}$	$\left\langle 50,49\right\rangle$	0(.)xxx...x1	[$2^{-49}$,1.0)	$b_{-1}b_{-2}...b_{-15}b_{-16}$
$x_n^{(1)}$	$\left\langle 50,49\right\rangle$	0(.)xxx...x1	[$2^{-49}$,1.0)	$b_{-1}b_{-2}...b_{-15}b_{-16}$

**Table 4:** Chosen bit arrangement of $Y_2$ to construct amounts of perturbation for control parameters of the PCM.

Parameter	Bit arrangement $Y_i$
$\delta _{\gamma ^{(2)}_n}$	$Y^{(a)}_2=\left[ (2,49)(1,48)B_0(2,47)(1,46)...(1,34)B_0B_0...B_0\right]$
$\delta _{\gamma ^{(1)}_n}$	$Y^{(b)}_2=\left[ (1,49)(2,48)B_0(1,47)(2,46)...(2,34)B_0B_0...B_0\right]$

**Table 5:** Chosen initial values.

Entity	Parameter	Value
Cat map	$\gamma ^{(2)}_n$	5.1122334455
Cat map	$\gamma ^{(1)}_n$	3.1122334455
Cat map	$x^{(2)}_0$	0.5544332211
Cat map	$x^{(1)}_0$	0.1122334456
SKS	$S_0$	0x12345678
BPP	$p^{(0)}_k$, $k=1,...,8$	[0xAC, 0x53BD01, 0x7F05F2, 0x9EC7AD, 0x1A, 0x05D4CB, 0xF0, 0xE8]
BPP	$c^{(0)}_k$, $k=1,...,8$	[0xE4, 0xDE2F91, 0x57CD1A, 0xA0D14F, 0xC8, 0x1B6AA3, 0xCD, 0x9B]

**Table 6:** Bit positions of $X_R$ to produce pseudo-random values.

Value of	Bits of $x_n^{(i)}$	*k*	Chosen			
			24 bits for $bs^{(S)}_k$	24 bits for $bs^{(D)}_k$	10 bits for $bs^{(I)}_k$	10 bits for $bs^{(J)}_k$
$x_n^{(2)}$	$b_{-17}...b_{-47}$	1	$b_{-17}...b_{-40}$	$b_{-18}...b_{-41}$	$b_{-31}...b_{-40}$	$b_{-32}...b_{-41}$
		2	$b_{-19}...b_{-42}$	$b_{-20}...b_{-43}$	$b_{-33}...b_{-42}$	$b_{-34}...b_{-43}$
		3	$b_{-21}...b_{-44}$	$b_{-22}...b_{-45}$	$b_{-35}...b_{-44}$	$b_{-36}...b_{-45}$
		4	$b_{-23}...b_{-46}$	$b_{-24}...b_{-47}$	$b_{-37}...b_{-46}$	$b_{-38}...b_{-47}$
$x_n^{(1)}$	$b_{-18}...b_{-48}$	5	$b_{-18}...b_{-41}$	$b_{-19}...b_{-42}$	$b_{-32}...b_{-41}$	$b_{-33}...b_{-42}$
		6	$b_{-20}...b_{-43}$	$b_{-21}...b_{-44}$	$b_{-34}...b_{-43}$	$b_{-35}...b_{-44}$
		7	$b_{-22}...b_{-45}$	$b_{-23}...b_{-46}$	$b_{-36}...b_{-45}$	$b_{-37}...b_{-46}$
		8	$b_{-24}...b_{-47}$	$b_{-25}...b_{-48}$	$b_{-38}...b_{-47}$	$b_{-39}...b_{-48}$

**Fig. 6:**
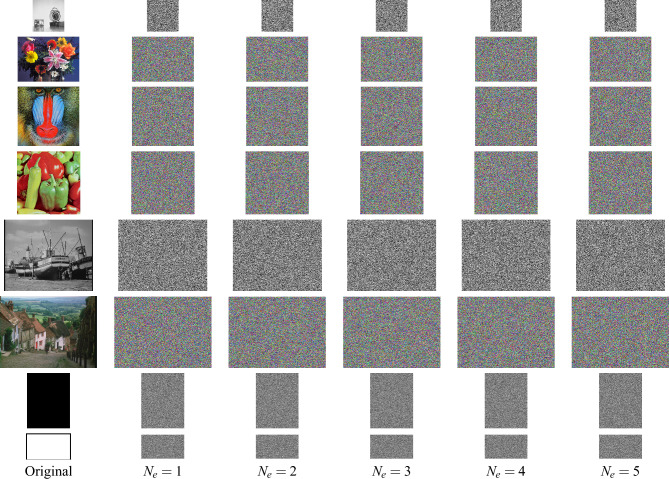
Ciphertext images in the example using Structure 1 in Fig. 1a encryption rounds $N_e=1,...,5$.

### Simulation results

**Fig. 7:**
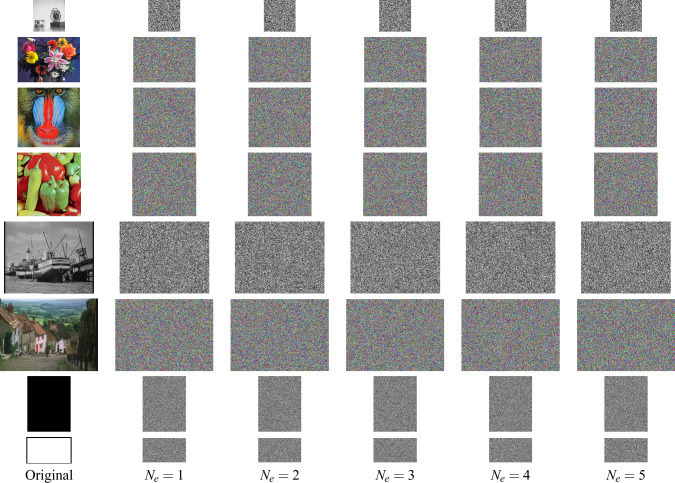
Ciphertext images in the example using Structure 2 in Fig. 1b encryption rounds $N_e=1,...,5$.

**Fig. 8:**
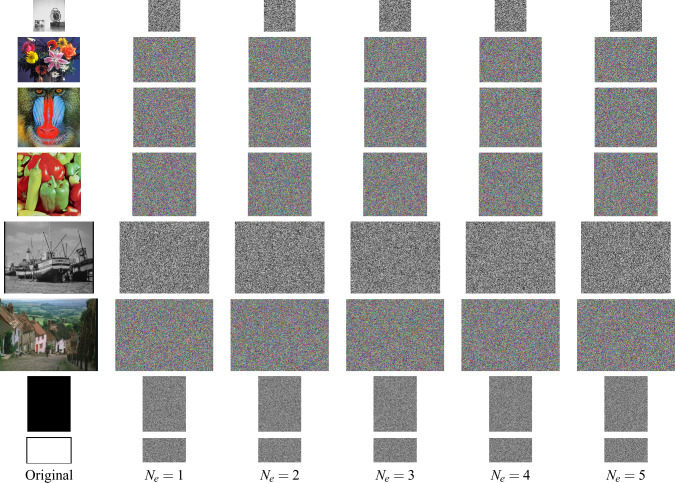
Ciphertext images in the example using Structure 3 in Fig. 1c encryption rounds $N_e=1,...,5$.

Here, the set of eight images is encrypted for several rounds of encryption, $n_k=1..N_e$, with $k=1...K$ and $N_e=5$. Figures 6, 7 and 8 display the ciphertext images produced by the exemplar designs using Structures 1, 2, and 3, respectively. The ciphertext images look like random images in every first round of encryption. In addition, these ciphertext images were successfully decrypted for any selected individual ciphered images.

It should be noted that the three-color image is considered as a grayscale image with a pixel depth of 24 bits. However, the color image is separated into three 8-bit grayscale images for analysis.

### Statistical analysis

Firstly, the randomness test is performed using the NIST SP 800-22 test standard to confirm the quality of session keys. Then, a statistical analysis of ciphertext images is performed with two main metrics, namely information entropy and correlation coefficients. Statistical analysis is measured for individual channels of color images, whereas bits of color channels are concatenated for encryption. Table 7 shows the information entropy and correlation coefficients of the original images.

**Table 7:** Statistical measures of original images.

	Entropy			Chi-square test ($\chi ^2$)			Correlation coefficients in								
							Horizontal			Vertical			Diagonal		
Channel	R	G	B	R	G	B	R	G	B	R	G	B	R	G	B
Baboon	6.501	6.446	6.273	579,798.39	595,896.90	742,573.87	0.924	0.869	0.907	0.874	0.776	0.884	0.867	0.750	0.854
Flowers	7.383	7.235	7.364	206,427.64	272706.08	192,953.29	0.972	0.951	0.953	0.972	0.950	0.953	0.955	0.922	0.926
Peppers	7.339	7.496	7.059	213,187.22	318,382.93	491,428.18	0.964	0.981	0.967	0.966	0.982	0.966	0.956	0.969	0.948
Goldhill	7.471	7.429	7.476	231332.11	268762.60	278677.24	0.968	0.973	0.977	0.961	0.977	0.979	0.940	0.958	0.962
Black	0.000			124,093,200.00			NaN			NaN			NaN		
Boats	7.088			586,046.22			0.969			0.978			0.950		
Clock	6.706			282061.56			0.956			0.974			0.939		
White	0.000			109,630,537.22			NaN			NaN			NaN		

#### NIST randomness test for session keys

Here, the randomness test is performed for the bit sequences generated by the SKS blocks to confirm the quality of the session keys. As shown in Fig. 1, the session keys are $sk_k$ with $k=1,2,\cdots ,8$, and more than 2 million bits of each session key $sk_k$ are recorded for the test. In this study, the NIST SP 800-22 test standard is used for the session key randomness test. Each session key $sk_k$ with a length of 165.457.600 bits is collected during encryption for the test. The test result is judged by $P-value$, and it is passed if $P-value>0.01$. Table 8 shows the test result of $sk_k$ with $k=1,2,\cdots ,8$, and it is clear that all $P-value$ are greater than 0.01 or pass. In other words, all the session keys in the exemplar simulation have good randomness and are qualified for encryption.

**Table 8:** The result of NIST test.

Sub-tests	$sk_1$	$sk_2$	$sk_3$	$sk_4$	$sk_5$	$sk_6$	$sk_7$	$sk_8$	Result
Frequency	0.3505	0.9114	0.3505	0.0179	0.3505	0.7399	0.5341	0.1223	All Pass
Block Frequency (m=128)	0.7399	0.5341	0.3505	0.7399	0.3505	0.5341	0.1223	0.3505	All Pass
Cusum-Forward	0.5341	0.3505	0.3505	0.1223	0.2133	0.1223	0.5341	0.7399	All Pass
Cusum-Reverse	0.9114	0.3505	0.5341	0.1223	0.3505	0.3505	0.5341	0.1223	All Pass
Runs	0.9915	0.1223	0.7399	0.7399	0.3505	0.3505	0.2133	0.3505	All Pass
Longest Runs of Ones	0.7399	0.1223	0.5341	0.5341	0.2133	0.5341	0.9114	0.7399	All Pass
Rank	0.2133	0.1223	0.2133	0.7399	0.3505	0.0179	0.5341	0.0179	All Pass
FFT	0.5341	0.7399	0.3505	0.1223	0.5341	0.9915	0.9114	0.3505	All Pass
NonOverlapping Templates ($m=9$)	0.4923	0.5030	0.5424	0.4888	0.4684	0.5091	0.4672	0.4534	All Pass
Overlapping Templates ($m=9$)	0.3505	0.7399	0.9114	0.0669	0.7399	0.0352	0.5341	0.5341	All Pass
Universal	0.3353	0.4584	0.3655	0.5486	0.2255	0.5423	0.0313	0.4896	All Pass
Approximate Entropy ($m=10$)	0.7399	0.9114	0.5341	0.5341	0.5341	0.3505	0.9915	0.3505	All Pass
Serial ($m=16$) $P-value_1$	0.0669	0.9114	0.1223	0.2133	0.3505	0.5341	0.2133	0.1223	All Pass
Serial ($m=16$) $P-value_2$	0.5341	0.5341	0.2133	0.2133	0.7399	0.7399	0.9114	0.3505	All Pass
Linear Complexity ($M=500$)	0.2133	0.2133	0.9114	0.7399	0.1223	0.0089	0.1223	0.2133	All Pass

#### Information entropy

The information entropy (*IE*) of an image measures the randomness of the values of the pixels, and the ideal value of *IE* is equal to the number of bits that represent the pixels. An image is mostly random if its *IE* is close to the ideal value.

In this example, *IE* of the color images is computed for each channel, so the ideal value of *IE* is 8 bits. Table 9 displays *IE* of encrypted images with different numbers of encryption rounds, $N_e=1,...,5$. The values of *IE* of all encrypted images are almost equal to the ideal value (8 bits) in every encryption round. The information entropy of all plaintext images in the simulation changes quickly from small values in the original images as given in Table 7 to almost 8 after the first round of encryption, at $N_e=1$. The average information entropy is measured in all ciphertext images and is unchanged with increasing number of encryption rounds. In all simulations, the average value of 7.9992 is very close to the ideal value of 8, which means that the values of the pixels in the ciphertext images are highly random and uniformly distributed.

**Table 9:** Information entropy of encrypted images.

Exemplar design using Structure 1 in Fig. 1a															
$N_e$	1			2			3			4			5		
Channel	R	G	B	R	G	B	R	G	B	R	G	B	R	G	B
Baboon	7.9993	7.9992	7.9992	7.9991	7.9992	7.9993	7.9992	7.9993	7.9992	7.9992	7.9992	7.9993	7.9992	7.9992	7.9993
Flowers	7.9989	7.9990	7.9991	7.9990	7.9990	7.9989	7.9989	7.9990	7.9990	7.9989	7.9989	7.9990	7.9989	7.9989	7.9990
Peppers	7.9994	7.9993	7.9992	7.9994	7.9992	7.9993	7.9993	7.9994	7.9992	7.9994	7.9994	7.9993	7.9992	7.9994	7.9992
Goldhill	7.9996	7.9996	7.9996	7.9996	7.9996	7.9996	7.9996	7.9996	7.9996	7.9996	7.9996	7.9996	7.9996	7.9995	7.9996
Black	7.9996			7.9996			7.9996			7.9996			7.9996		
Boats	7.9996			7.9996			7.9996			7.9995			7.9995		
Clock	7.9974			7.9972			7.9971			7.9970			7.9970		
White	7.9995			7.9996			7.9995			7.9996			7.9996		
Average	7.9992			7.9992			7.9992			7.9992			7.9992		
Exemplar design using Structure 2 in Fig. 1b															
$N_e$	1			2			3			4			5		
Channel	R	G	B	R	G	B	R	G	B	R	G	B	R	G	B
Baboon	7.9993	7.9992	7.9992	7.9993	7.9992	7.9992	7.9993	7.9993	7.9992	7.9992	7.9992	7.9991	7.9991	7.9993	7.9993
Flowers	7.9989	7.9992	7.9988	7.9990	7.9989	7.9990	7.9988	7.9991	7.9990	7.9989	7.9991	7.9990	7.9990	7.9989	7.9991
Peppers	7.9993	7.9992	7.9994	7.9993	7.9994	7.9994	7.9993	7.9992	7.9993	7.9994	7.9993	7.9993	7.9992	7.9994	7.9994
Goldhill	7.9996	7.9996	7.9996	7.9996	7.9996	7.9996	7.9996	7.9996	7.9996	7.9996	7.9996	7.9995	7.9995	7.9996	7.9996
Black	7.9996			7.9996			7.9996			7.9997			7.9997		
Boats	7.9996			7.9995			7.9996			7.9995			7.9996		
Clock	7.9974			7.9976			7.9973			7.9972			7.9970		
White	7.9996			7.9996			7.9996			7.9996			7.9996		
Average	7.9992			7.9992			7.9992			7.9992			7.9992		
Exemplar design using Structure 3 in Fig. 1c															
$N_e$	1			2			3			4			5		
Channel	R	G	B	R	G	B	R	G	B	R	G	B	R	G	B
Baboon	7.9992	7.9993	7.9992	7.9992	7.9992	7.9992	7.9992	7.9992	7.9993	7.9993	7.9992	7.9992	7.9992	7.9992	7.9992
Flowers	7.9991	7.9988	7.9991	7.9991	7.9990	7.9989	7.9988	7.9991	7.9990	7.9992	7.9991	7.9991	7.9989	7.9990	7.9991
Peppers	7.9993	7.9994	7.9993	7.9992	7.9993	7.9994	7.9993	7.9994	7.9994	7.9993	7.9994	7.9993	7.9992	7.9993	7.9993
Goldhill	7.9996	7.9996	7.9997	7.9996	7.9996	7.9996	7.9996	7.9996	7.9997	7.9996	7.9996	7.9997	7.9996	7.9996	7.9996
Black	7.9997			7.9996			7.9996			7.9996			7.9996		
Boats	7.9995			7.9996			7.9995			7.9995			7.9995		
Clock	7.9974			7.9972			7.9976			7.9979			7.9974		
White	7.9996			7.9996			7.9996			7.9996			7.9996		
Average	7.9992			7.9992			7.9992			7.9993			7.9992		

#### Histogram analysis

The histogram of an image reflects the distribution of pixel values, and the statistical histogram can be measured using the chi-square test with a significance level of 0.05. The equation of $\chi ^2$ is

18 $$\begin{aligned} \chi ^2=\sum _{i=0}^{V-1} \frac{\left( O_i-E_i\right) ^2}{E_i} \end{aligned}$$

where *V* is the number of gray level ($V = 256$ for 8-bit grayscale images), and $O_i$ and $E_i$ are respectively observed and expected occurrence frequencies of gray level *i*, with $0\le i\le V-1$. The expected occurrence frequency of 8-bit grayscale images is $E_i = \frac{M*N}{V}$; $M\times N$ is the size of the images. The hypothesis test considers the significance of the histogram if it complies with a uniform distribution, and the test is accepted (or the histogram is uniformly distributed) if $\chi ^2\le \chi ^2_\alpha (V-1)$. In this example, the significance level $\alpha = 0.05$ is considered, and $\chi ^2_{0.05}(255) = [293.247]$.

Table 10 gives the test results of the ciphertext images for the exemplar simulation. The value of $\chi ^2$ is less than [!*tb*]. In other words, the histogram statistics of all ciphertext images pass the test, or the histogram of ciphertext images is uniformly distributed. It is clear that the chi-square values of the plain text images in Table 7 are large, but are quickly reduced to and smaller than $\chi ^2_{0.05}(255)$. The average values are calculated on the overall ciphertext images, and the chi-square test with significant level $\alpha = 0.05$ and passed, even after the first encryption round, means that the histogramof ciphertext images are uniformly distributed.

**Table 10:** Chi-square test results.

$\chi ^2$ of encrypted images in the exemplar simulation using Structure 1 in Fig. 1a															
$N_e$	1			2			3			4			5		
Channel	R	G	B	R	G	B	R	G	B	R	G	B	R	G	B
Baboon	225.024	274.968	274.831	283.294	278.586	238.726	270.159	236.399	276.024	266.733	272.489	244.002	278.942	256.361	231.366
Flowers	287.042	241.166	229.438	248.549	261.338	287.673	266.248	251.737	252.365	264.305	277.905	251.847	266.132	276.033	250.014
Peppers	271.232	246.325	226.382	272.219	241.743	227.020	212.012	246.864	245.899	256.662	267.964	239.662	269.368	245.315	271.356
Goldhill	256.172	223.146	269.022	255.493	268.755	258.332	225.218	269.318	278.298	227.722	253.505	252.721	241.462	284.386	258.382
Black	258.190			275.968			266.930			242.293			266.213		
Boats	232.903			244.562			246.557			264.909			299.651		
Clock	237.016			251.578			260.742			272.211			270.281		
White	267.814			262.724			283.123			258.653			215.717		
Average	251.292 (Pass)			259.785 (Pass)			255.493 (Pass)			257.099 (Pass)			261.311 (Pass)		

$\chi ^2$ of encrypted images in the exemplar simulation using Structure 2 in Fig. 1b															
$N_e$	1			2			3			4			5		
Channel	R	G	B	R	G	B	R	G	B	R	G	B	R	G	B
Baboon	226.044	260.122	260.563	236.239	277.043	263.742	248.465	235.881	274.929	253.086	251.480	301.811	286.812	227.428	234.404
Flowers	267.646	205.408	292.405	248.334	287.783	259.227	295.285	231.308	252.419	273.642	233.257	262.540	243.087	282.856	227.670
Peppers	263.356	269.438	255.138	234.802	251.378	280.429	220.387	260.395	249.479	255.202	249.676	290.457	270.369	251.735	220.364
Goldhill	266.360	269.895	268.893	280.378	249.177	232.245	280.120	252.197	253.164	273.825	235.658	294.107	304.127	255.396	238.926
Black	241.076			251.418			276.914			230.526			216.151		
Boats	233.224			262.757			250.315			261.549			230.815		
Clock	240.406			222.055			244.469			255.430			273.727		
White	211.514			240.997			238.493			261.427			245.593		
Average	251.968 (Pass)			254.875 (Pass)			254.014 (Pass)			261.480 (Pass)			250.591 (Pass)		

$\chi ^2$ of encrypted images in the exemplar simulation using Structure 3 in Fig. 1c															
$N_e$	1			2			3			4			5		
Channel	R	G	B	R	G	B	R	G	B	R	G	B	R	G	B
Baboon	258.603	235.392	280.506	253.197	276.450	265.444	259.392	251.960	233.137	243.712	258.878	274.037	269.745	261.978	270.443
Flowers	235.692	296.968	220.527	219.684	245.689	273.235	295.500	219.682	262.359	205.923	232.756	231.313	267.801	254.817	233.418
Peppers	265.548	249.135	266.452	220.354	227.686	267.629	255.489	275.327	256.135	247.028	255.613	239.657	228.358	273.516	265.902
Goldhill	231.418	249.874	218.995	222.326	247.528	237.083	267.491	278.048	203.367	241.728	271.877	219.586	245.952	265.682	253.891
Black	203.720			245.266			267.113			266.415			256.545		
Boats	281.506			257.516			273.678			286.019			263.912		
Clock	237.133			255.656			218.313			186.922			234.008		
White	235.127			247.135			258.162			240.050			259.806		
Average	247.912 (Pass)			247.617 (Pass)			254.697 (Pass)			243.845 (Pass)			256.611 (Pass)		

#### Correlation coefficients between adjacent pixels

It is well known that a high similarity in the values of adjacent pixels in an image makes the visual structure. In this example, the correlation coefficients (CCs) between pairs of adjacent pixels in three directions, that is, horizontal (*H*), vertical (*V*) and diagonal (*D*), is measured, and the Pearson correlation coefficient is used for measurements for grayscale images and every channel of color images. The closer the CCs are to zero, the more random the image is.

Tables 11, 12 and 13 show the CCs of the ciphertext images in the exemplar simulation of each design. The results show that the CC values are close to zero in every encryption round, which means that the ciphertext images. However, the values of Clock’s CCs are the biggest in all examples. The average values are calculated on all the ciphertext images of the cohort. Even the average values are small, but they fluctuate around zero and regardless to number of encryption rounds. This means that the visual structure in the plaintext images, with large value of CCs as seen in Table 7, is destroyed in the ciphertext images, with small values in all directions of horizontal, vertical and diagonal, as seen in Table 11, 12 and 13.

Compared with the values of the original images given in Table 7, the IE, the Chi-square test and the CC of the plain images are significantly changed compared to those of the ciphertext images given in Table 9, 10,  11, 12 and 13.

**Table 11:** Correlation coefficients of encrypted images in the exemplar design using Structure 1 in Fig. 1a.

Correlation coefficients in horizontal															
$N_e$	1			2			3			4			5		
Channel	R	G	B	R	G	B	R	G	B	R	G	B	R	G	B
Baboon	0.0010	-0.0008	-0.0027	0.0025	0.0013	-0.0002	-0.0017	-0.0006	0.0027	-0.0008	-0.0032	0.0011	-0.0014	-0.0022	0.0004
Flowers	0.0011	0.0005	-0.0023	0.0018	0.0005	-0.0011	0.0028	0.0004	-0.0009	0.0074	0.0063	0.0031	0.0017	0.0017	-0.0020
Peppers	0.0012	-0.0002	0.0003	-0.0011	-0.0009	-0.0035	0.0017	0.0008	0.0006	-0.0010	-0.0004	-0.0012	0.0001	-0.0001	-0.0003
Goldhill	0.0017	0.0004	-0.0028	-0.0022	-0.0002	-0.0010	0.0000	0.0024	-0.0021	-0.0033	-0.0008	0.0015	0.0035	0.0008	0.0013
Black	-0.0007			0.0000			-0.0011			-0.0008			-0.0018		
Boats	-0.0004			0.0000			0.0000			-0.0003			0.0001		
Clock	0.0033			0.0013			0.0043			-0.0054			-0.0050		
White	0.0018			0.0001			-0.0003			-0.0003			0.0021		
Average	0.0001			-0.0002			0.0006			0.0001			-0.0001		

Correlation coefficients in vertical															
$N_e$	1			2			3			4			5		
Channel	R	G	B	R	G	B	R	G	B	R	G	B	R	G	B
Baboon	-0.0009	0.0017	-0.0052	-0.0022	0.0018	-0.0020	0.0018	-0.0015	0.0002	-0.0005	0.0014	0.0011	-0.0010	0.0025	0.0001
Flowers	0.0016	0.0004	-0.0022	0.0009	0.0011	0.0017	-0.0010	-0.0022	0.0029	0.0005	-0.0061	0.0006	0.0013	-0.0041	-0.0011
Peppers	-0.0013	-0.0006	-0.0002	-0.0003	-0.0008	-0.0005	0.0003	-0.0006	0.0006	-0.0001	-0.0016	-0.0016	0.0003	-0.0009	-0.0008
Goldhill	0.0012	-0.0003	0.0002	0.0025	-0.0006	-0.0003	0.0014	-0.0032	-0.0004	0.0016	-0.0009	-0.0025	-0.0024	-0.0010	0.0017
Black	0.0016			0.0041			-0.0009			-0.0008			-0.0003		
Boats	-0.0011			-0.0020			0.0029			-0.0019			-0.0006		
Clock	0.0035			-0.0053			-0.0022			0.0001			-0.0020		
White	0.0000			0.0005			0.0022			0.0002			0.0000		
Average	-0.0005			0.0001			0.0000			-0.0007			-0.0005		

Correlation coefficients in diagonal															
$N_e$	1			2			3			4			5		
Channel	R	G	B	R	G	B	R	G	B	R	G	B	R	G	B
Baboon	-0.0018	0.0019	-0.0035	-0.0026	0.0012	-0.0012	-0.0009	-0.0039	-0.0007	-0.0038	-0.0026	0.0015	0.0016	-0.0027	-0.0016
Flowers	0.0043	-0.0011	-0.0018	0.0025	-0.0005	0.0006	-0.0062	0.0007	-0.0019	0.0020	-0.0046	-0.0025	-0.0031	-0.0017	-0.0001
Peppers	0.0003	-0.0002	-0.0012	0.0002	-0.0004	-0.0005	-0.00031	0.0033	-0.0001	0.0012	-0.0006	0.0004	-0.0012	-0.0001	0.0009
Goldhill	0.0002	-0.0004	0.0005	-0.0016	0.0020	0.0016	-0.0013	-0.0002	-0.0009	-0.0004	0.0005	-0.0006	-0.0020	0.0006	0.0013
Black	-0.0021			-0.0004			0.0000			0.0026			-0.0004		
Boats	-0.0002			-0.0014			-0.0014			0.0000			0.0014		
Clock	0.0013			0.0027			-0.0022			-0.0015			-0.0003		
White	-0.0044			-0.0007			0.0009			0.0012			0.0012		
Average	-0.0005			0.0001			-0.0009			-0.0005			-0.0004

**Table 12:** Correlation coefficients of encrypted images in the exemplar design using Structure 2 in Fig. 1b.

Correlation coefficients in horizontal															
$N_e$	1			2			3			4			5		
Channel	R	G	B	R	G	B	R	G	B	R	G	B	R	G	B
Baboon	-0.0007	-0.0006	-0.0022	-0.0036	0.0001	0.0024	-0.0008	0.0029	0.0025	0.0048	-0.0020	0.0004	-0.0010	-0.0006	-0.0020
Flowers	0.0046	0.0017	0.0016	-0.0026	0.0012	0.0009	0.0040	-0.0003	0.0013	0.0002	0.0003	0.0016	-0.0027	0.0020	-0.0022
Peppers	0.0007	0.0014	-0.0006	-0.0009	-0.0015	0.0016	0.0005	-0.0020	0.0016	-0.0029	-0.0001	-0.0006	-0.0001	0.0002	0.0002
Goldhill	0.0004	-0.0007	0.0007	-0.0014	0.0008	-0.0004	0.0001	-0.0037	-0.0002	0.0028	-0.0027	-0.0007	0.0010	0.0029	0.0017
Black	0.0007			-0.0010			-0.0009			-0.0012			0.0002		
Boats	-0.0024			-0.0018			-0.0006			-0.0015			-0.0005		
Clock	0.0013			0.0084			0.0018			0.0071			-0.0012		
White	0.0021			0.0019			-0.0033			0.0027			-0.0005		
Average	0.0005			0.0003			0.0002			0.0005			-0.0002		

Correlation coefficients in vertical															
$N_e$	1			2			3			4			5		
Channel	R	G	B	R	G	B	R	G	B	R	G	B	R	G	B
Baboon	-0.0002	0.0000	0.0028	0.0009	-0.0040	-0.0005	-0.0029	0.0008	0.0022	-0.0053	-0.0003	0.0017	-0.0011	-0.0027	-0.0022
Flowers	0.0020	-0.0021	-0.0024	0.0009	0.0037	-0.0047	0.0011	-0.0013	0.0046	0.0000	-0.0004	0.0023	0.0016	0.0051	-0.0015
Peppers	-0.0003	-0.0004	0.0005	-0.0002	-0.0005	0.0004	0.0009	0.0014	0.0003	-0.0010	0.0026	0.0009	-0.0001	0.0023	-0.0029
Goldhill	-0.0005	0.0008	-0.0009	0.0003	0.0010	0.0002	-0.0019	0.0002	-0.0010	-0.0020	-0.0013	0.0014	0.0025	-0.0023	0.0001
Black	0.0008			0.0009			-0.0002			0.0000			0.0001		
Boats	0.0020			0.0018			0.0008			-0.0002			-0.0017		
Clock	-0.0017			0.0011			0.0041			-0.0025			0.0086		
White	0.0007			0.0022			-0.0021			0.0004			-0.0007		
Average	0.0001			0.0002			0.0004			-0.0002			0.0003		

Correlation coefficients in diagonal															
$N_e$	1			2			3			4			5		
Channel	R	G	B	R	G	B	R	G	B	R	G	B	R	G	B
Baboon	0.0012	0.0009	-0.0013	-0.0007	-0.0005	0.0013	-0.0002	-0.0018	0.0007	0.0003	-0.0028	-0.0005	-0.0005	0.0004	-0.0013
Flowers	0.0012	0.0030	-0.0021	-0.0022	0.0006	0.0012	0.0018	-0.0031	0.0050	0.0049	-0.0057	0.0016	0.0036	-0.0012	-0.0021
Peppers	0.0019	0.0015	0.0022	-0.0004	0.0001	-0.0009	-0.0002	-0.0017	0.0002	0.0004	-0.0015	0.0009	0.0007	-0.0002	0.0004
Goldhill	-0.0006	-0.0031	0.0011	0.0008	-0.0003	0.0007	0.0014	0.0007	-0.0007	-0.0010	0.0003	-0.0038	0.0000	-0.0039	0.0011
Black	-0.0026			-0.0019			0.0016			-0.0023			0.0006		
Boats	-0.0006			-0.0007			-0.0015			-0.0005			-0.0011		
Clock	-0.0122			0.0027			-0.0021			-0.0013			-0.0032		
White	-0.0002			0.0005			0.0012			0.0024			0.0023		
Average	-0.0006			0.0000			0.0001			-0.0005			-0.0003

**Table 13:** Correlation coefficients of encrypted images in the exemplar design using Structure 3 in Fig. 1c.

Correlation coefficients in horizontal															
$N_e$	1			2			3			4			5		
Channel	R	G	B	R	G	B	R	G	B	R	G	B	R	G	B
Baboon	0.0019	0.0003	0.0015	0.0017	-0.0014	0.0005	0.0001	-0.0031	-0.0010	-0.0017	0.0001	-0.0007	-0.0033	0.0016	0.0004
Flowers	0.0040	0.0000	0.0008	-0.0007	0.0005	0.0048	0.0000	-0.0019	-0.0039	-0.0002	0.0025	-0.0040	-0.0015	-0.0024	-0.0045
Peppers	0.0002	-0.0017	0.0009	-0.0019	0.0003	0.0011	0.0003	0.0009	-0.0028	-0.0008	0.0016	-0.0042	0.0006	-0.0007	0.0003
Goldhill	0.0032	0.0017	0.0003	-0.0016	-0.0002	-0.0021	-0.0018	-0.0003	0.0005	0.0004	0.0001	-0.0007	0.0028	0.0010	0.0002
Black	-0.0007			0.0024			0.0023			0.0006			-0.0017		
Boats	0.0003			0.0007			-0.0003			0.0015			-0.0041		
Clock	-0.0027			-0.0025			0.0072			0.0067			-0.0027		
White	-0.0012			0.0000			-0.0024			-0.0001			0.0004		
Average	0.0006			0.0001			-0.0004			0.0001			-0.0009		
Correlation coefficients in vertical															
$N_e$	1			2			3			4			5		
Channel	R	G	B	R	G	B	R	G	B	R	G	B	R	G	B
Baboon	0.0003	-0.0028	0.0003	0.0031	-0.0011	0.0018	0.0007	0.0014	0.0014	0.0020	0.0013	-0.0012	-0.0025	-0.0006	0.0002
Flowers	0.0013	0.0047	0.0046	0.0013	-0.0032	0.0003	-0.0043	-0.0008	0.0049	-0.0009	0.0022	0.0011	-0.0002	-0.0008	-0.0035
Peppers	0.0015	-0.0011	-0.0001	0.0008	-0.0019	0.0016	0.0025	0.0014	0.0018	-0.0004	0.0011	-0.0020	0.0017	0.0001	0.0002
Goldhill	0.0001	0.0014	-0.0024	0.0010	-0.0021	0.0026	0.0029	-0.0022	-0.0026	-0.0003	0.0005	-0.0023	-0.0008	0.0001	0.0007
Black	0.0006			0.0000			-0.0009			-0.0010			-0.0007		
Boats	0.0002			-0.0006			0.0014			-0.0003			-0.0021		
Clock	0.0069			-0.0030			0.0003			0.0031			0.0056		
White	-0.0010			0.0020			-0.0005			-0.0014			-0.0005		
Average	0.0009			0.0002			0.0005			0.0001			-0.0002		
Correlation coefficients in diagonal															
$N_e$	1			2			3			4			5		
Channel	R	G	B	R	G	B	R	G	B	R	G	B	R	G	B
Baboon	0.0008	0.0034	0.0015	-0.0015	0.0000	-0.0002	0.0003	0.0019	-0.0009	0.0012	-0.0018	0.0012	-0.0031	0.0006	0.0003
Flowers	-0.0024	0.0033	0.0055	0.0006	-0.0011	0.0000	-0.0003	-0.0018	0.0000	0.0007	0.0042	0.0056	0.0025	0.0015	-0.0057
Peppers	-0.0011	-0.0026	-0.0005	-0.0019	0.0004	0.0018	-0.0002	-0.0014	-0.0004	0.0017	-0.0020	-0.0016	0.0017	-0.0005	0.0003
Goldhill	-0.0026	0.0004	-0.0016	0.0009	-0.0006	0.0019	-0.0006	-0.0008	0.0006	-0.0008	-0.0013	-0.0007	-0.0006	-0.0004	0.0007
Black	0.0015			0.0008			-0.0027			0.0027			0.0005		
Boats	0.0013			-0.0007			0.0010			0.0005			0.0029		
Clock	-0.0067			0.0027			0.0003			-0.0002			-0.0017		
White	-0.0011			-0.0020			0.0000			0.0004			0.0014		
Average	-0.0001			0.0001			-0.0003			0.0006			0.0000		

### Security analysis

Let us consider the security aspects of the examples using the proposed structures using two crucial metrics, namely the secret key space and the sensitivity of the secret key.

#### Space of the secret key

The space of the secret key is considered by the number of flippable bits which are initial values of parameters. In this simulation, the flippable bits of initial values of the Cat map ($X_0$ and $\Gamma _0$) and of the SKS ($S_0$) as well as of plaintext and ciphertext pixels ($c^{(0)}_k$ and $p^{(0)}_k$ with $k=1..K$) in the context of encrypting eight images. Table 14 shows 486 flippable bits that contribute by parameters. In other words, the secret key space is $2^{486}$, and the ciphers in this exemplar simulation based on the proposed structures can resist brute-force attacks using modern computers.

**Table 14:** Number of bits for the secret key.

Entity	Parameters	Number of flipplable bits (bits)	Reference
Cat map	$\gamma ^{(2)}_0$	51	Eq. (17) and Table 3 and 5
Cat map	$\gamma ^{(1)}_0$	51	Eq. (17) and Table 3 and 5
Cat map	$x^{(2)}_0$	48	Eq. (17) and Table 3 and 5
Cat map	$x^{(1)}_0$	48	Eq. (17) and Table 3 and 5
Initial value of SKS	$S_0$	32	Eq. (12) and Table 5
Initial values of ciphertext	$\{c^{(0)}_1, c^{(0)}_2,..., c^{(0)}_K\}$	$\sum _{k=1}^{K=8} q_k$=128	Table 1
Initial values of plaintext	$\{p^{(0)}_1, p^{(0)}_2,..., p^{(0)}_K\}$	$\sum _{k=1}^{K=8} q_k$=128	Table 1
Sum		486	

#### Sensitivity of the secret key

The sensitivity of the secret key of a MIE algorithm can be assessed by measuring the difference between two sets of ciphertext images that are obtained from the same set of plain images using two almost identical secret keys. Two secret keys are said to be almost identical because one of them is produced by using the other with slight modification. In this simulation, the least significant bits of the parameters are changed in turn to make the smallest difference between pairs of secret keys, as shown in Table 15.

**Table 15:** Modified secret key for the sensitivity analysis of secret key.

Parameters	Bit being changed	amount of difference	Reference/Note
$\gamma ^{(2)}_0$	$b_{-49}$	$2^{-49}$	Table 3
$\gamma ^{(1)}_0$	$b_{-49}$	$2^{-49}$	Table 3
$x^{(2)}_0$	$b_{-48}$	$2^{-48}$	Table 3
$x^{(1)}_0$	$b_{-48}$	$2^{-48}$	Table 3
$S_0$	$b_{0}$	1	Table 5
$\{c^{(0)}_1, c^{(0)}_2,..., c^{(0)}_K\}$	$b_{0}$	1	- Only one of initial ciphertext pixels changed
$\{p^{(0)}_1, p^{(0)}_2,..., p^{(0)}_K\}$	$b_{0}$	1	- Only one of initial plain pixels changed

The number of pixels change rate (NPCR) and the unified averaged changed intensity (UACI)^74,75^ are used to measure the sensitivity of the secret key in two sets of ciphertext images. Specifically, a pair of ciphertext images $C_1$ and $C_2$ are used to calculate NPCR and UACI as

19 $$\begin{aligned} NPCR=\frac{\sum _{x,y} D(x,y)}{M\times N}\times 100\%, \end{aligned}$$

and

20 $$\begin{aligned} UACI=\frac{1}{N^2}\left[ \sum _{x,y} \frac{C_1(x,y)-C_2(x,y)|}{255}\right] \times 100\%, \end{aligned}$$

where $D(x,y)=1$ if $C_1(x,y)\ne C_2(x,y)$, and $D(x,y)=0$ if $C_1(x,y)=C_2(x,y)$. The values of *NPCR* and *UACI* are used to determine the random tests. A random test with the critical value $\alpha =0.05$ is passed if $NPCR\ge 99.569\%$ and $UACI\in [33.282\%, 33.644\%]$ as mentioned in^75^.

As given in Eq. (16), the initial plain pixels, $p_k^{(0)}$ with $k=1,..,K$, are used to perturb the chaotic map in encryption for the last pixels in the final encryption round. So, the sensitivity of the initial plain pixels is not used to consider the sensitivity for encryption. Similarity, sensitivity of the initial ciphertext pixels is only considered for the decryption.

Tables 16, 17 18,  19, 20 and 21 present *NPCR* and *UACI* ciphertexts to analyze the sensitivity of each secret key parameter. The tests are measured for individual images and for all images using average values. It is clear that most of the tests for individual images have been passed, and average values indicate that cipherext images of the cohort have been passed for sensitivity of every parameter. Passing the test of average values is very important for the case of MIE. Here, the average values of *NPCR* and *UACI* fluctuate slightly around 99.6 and 33.4 for all changes in the secret key and regardless of the number of encryption rounds. The sensitivity test is passed for every parameter of the secret key; therefore, any parameters can be used equivalently to lengthen the number of bits of the secret key. In other words, the exemplar ciphers in this simulation that use the proposed structures are highly sensitive to the smallest changes in the secret key.

**Table 16:** Exemplar simulation for Structure 1 in Fig. 1a: Sensitivity of secret key by NPCR.

Sensitivity on $\gamma ^{(2)}_0$															
$N_e$	1			2			3			4			5		
Channel	R	G	B	R	G	B	R	G	B	R	G	B	R	G	B
Baboon	99.606	99.620	99.617	99.607	99.587	99.615	99.617	99.595	99.613	99.602	99.601	99.602	99.615	99.611	99.597
Flowers	99.606	99.603	99.610	99.610	99.604	99.587	99.610	99.617	99.586	99.622	99.599	99.601	99.597	99.603	99.610
Peppers	99.626	99.617	99.616	99.605	99.621	99.614	99.616	99.605	99.613	99.614	99.599	99.600	99.597	99.614	99.621
Goldhill	99.601	99.618	99.609	99.620	99.599	99.602	99.612	99.607	99.623	99.605	99.600	99.608	99.606	99.602	99.607
Black	99.613			99.619			99.612			99.592			99.614		
Boats	99.602			99.631			99.613			99.603			99.618		
Clock	99.635			99.661			99.611			99.599			99.622		
White	99.606			99.603			99.607			99.603			99.600		
Average	99.613 (Pass)			99.612 (Pass)			99.610 (Pass)			99.603 (Pass)			99.608 (Pass)		

Sensitivity on $\gamma ^{(1)}_0$															
$N_e$	1			2			3			4			5		
Channel	R	G	B	R	G	B	R	G	B	R	G	B	R	G	B
Baboon	99.625	99.592	99.625	99.608	99.602	99.607	99.590	99.639	99.610	99.604	99.598	99.627	99.614	99.593	99.615
Flowers	99.608	99.595	99.585	99.636	99.626	99.635	99.636	99.577	99.610	99.614	99.591	99.583	99.624	99.634	99.606
Peppers	99.615	99.618	99.620	99.600	99.605	99.625	99.617	99.620	99.618	99.609	99.616	99.634	99.626	99.617	99.595
Goldhill	99.602	99.616	99.615	99.608	99.604	99.631	99.605	99.619	99.614	99.606	99.601	99.596	99.617	99.595	99.612
Black	99.600			99.610			99.604			99.605			99.596		
Boats	99.603			99.616			99.588			99.611			99.615		
Clock	99.647			99.641			99.576			99.586			99.641		
White	99.607			99.610			99.604			99.598			99.618		
Average	99.611 (Pass)			99.617 (Pass)			99.608 (Pass)			99.605 (Pass)			99.614 (Pass)		

Sensitivity on $x^{(2)}_0$															
$N_e$	1			2			3			4			5		
Channel	R	G	B	R	G	B	R	G	B	R	G	B	R	G	B
Baboon	99.605	99.591	99.607	99.615	99.585	99.590	99.624	99.615	99.615	99.598	99.584	99.605	99.612	99.614	99.633
Flowers	99.584	99.632	99.593	99.616	99.608	99.627	99.613	99.605	99.606	99.601	99.615	99.604	99.598	99.626	99.619
Peppers	99.613	99.620	99.617	99.618	99.599	99.603	99.615	99.590	99.616	99.615	99.607	99.601	99.616	99.597	99.602
Goldhill	99.603	99.610	99.617	99.606	99.611	99.597	99.619	99.611	99.610	99.615	99.608	99.623	99.588	99.592	99.616
Black	99.625			99.617			99.605			99.607			99.607		
Boats	99.612			99.603			99.595			99.622			99.629		
Clock	99.576			99.635			99.606			99.612			99.617		
White	99.612			99.604			99.600			99.606			99.603		
Average	99.607 (Pass)			99.608 (Pass)			99.609 (Pass)			99.608 (Pass)			99.611 (Pass)		

Sensitivity on $x^{(1)}_0$															
$N_e$	1			2			3			4			5		
Channel	R	G	B	R	G	B	R	G	B	R	G	B	R	G	B
Baboon	99.606	99.591	99.628	99.605	99.605	99.602	99.594	99.611	99.611	99.624	99.602	99.598	99.610	99.605	99.598
Flowers	99.604	99.608	99.636	99.633	99.596	99.603	99.590	99.615	99.609	99.586	99.601	99.610	99.601	99.614	99.619
Peppers	99.621	99.609	99.610	99.622	99.618	99.624	99.609	99.602	99.608	99.612	99.623	99.597	99.593	99.620	99.611
Goldhill	99.617	99.609	99.614	99.619	99.612	99.609	99.619	99.626	99.605	99.612	99.628	99.592	99.610	99.596	99.612
Black	99.601			99.626			99.610			99.622			99.605		
Boats	99.612			99.584			99.621			99.606			99.613		
Clock	99.590			99.626			99.629			99.649			99.600		
White	99.610			99.609			99.606			99.596			99.615		
Average	99.610 (Pass)			99.612 (Pass)			99.610 (Pass)			99.610 (Pass)			99.608 (Pass)		

Sensitivity on $S_0$															
$N_e$	1			2			3			4			5		
Channel	R	G	B	R	G	B	R	G	B	R	G	B	R	G	B
Baboon	99.610	99.606	99.608	99.602	99.610	99.615	99.620	99.623	99.603	99.623	99.604	99.622	99.617	99.613	99.602
Flowers	99.599	99.609	99.588	99.602	99.599	99.602	99.613	99.622	99.628	99.617	99.607	99.608	99.570	99.622	99.592
Peppers	99.618	99.610	99.590	99.607	99.620	99.599	99.612	99.625	99.629	99.620	99.617	99.608	99.622	99.621	99.610
Goldhill	99.597	99.617	99.607	99.628	99.613	99.612	99.603	99.616	99.613	99.610	99.607	99.615	99.609	99.602	99.635
Black	99.618			99.618			99.605			99.590			99.604		
Boats	99.616			99.626			99.609			99.596			99.603		
Clock	99.602			99.657			99.594			99.574			99.580		
White	99.617			99.611			99.608			99.621			99.620		
Average	99.607 (Pass)			99.614 (Pass)			99.614 (Pass)			99.609 (Pass)			99.608 (Pass)

**Table 17:** Exemplar simulation for Structure 2 in Fig. 1b: Sensitivity of secret key by NPCR.

Sensitivity on $\gamma ^{(2)}_0$															
$N_e$	1			2			3			4			5		
Channel	R	G	B	R	G	B	R	G	B	R	G	B	R	G	B
Baboon	99.603	99.598	99.586	99.610	99.600	99.609	99.616	99.621	99.662	99.616	99.596	99.585	99.626	99.570	99.621
Flowers	99.599	99.627	99.609	99.596	99.609	99.641	99.573	99.604	99.627	99.614	99.596	99.612	99.596	99.608	99.589
Peppers	99.625	99.604	99.619	99.622	99.608	99.627	99.620	99.617	99.625	99.608	99.611	99.618	99.587	99.614	99.622
Goldhill	99.610	99.605	99.589	99.599	99.608	99.606	99.603	99.599	99.617	99.609	99.610	99.610	99.594	99.598	99.612
Black	99.615			99.607			99.614			99.607			99.621		
Boats	99.607			99.604			99.630			99.606			99.607		
Clock	99.576			99.620			99.615			99.596			99.588		
White	99.625			99.610			99.594			99.620			99.589		
Average	99.606 (Pass)			99.611 (Pass)			99.615 (Pass)			99.607 (Pass)			99.603 (Pass)		

Sensitivity on $\gamma ^{(1)}_0$															
$N_e$	1			2			3			4			5		
Channel	R	G	B	R	G	B	R	G	B	R	G	B	R	G	B
Baboon	99.601	99.618	99.622	99.615	99.617	99.602	99.615	99.630	99.606	99.595	99.607	99.626	99.591	99.621	99.613
Flowers	99.606	99.603	99.613	99.590	99.605	99.592	99.613	99.625	99.602	99.591	99.588	99.584	99.616	99.593	99.623
Peppers	99.621	99.609	99.615	99.587	99.621	99.615	99.599	99.604	99.613	99.621	99.606	99.614	99.618	99.620	99.611
Goldhill	99.607	99.599	99.612	99.595	99.619	99.614	99.614	99.615	99.592	99.606	99.605	99.612	99.612	99.589	99.613
Black	99.596			99.609			99.618			99.609			99.620		
Boats	99.614			99.590			99.605			99.623			99.607		
Clock	99.657			99.580			99.596			99.583			99.661		
White	99.602			99.612			99.626			99.602			99.610		
Average	99.612 (Pass)			99.604 (Pass)			99.611 (Pass)			99.605 (Pass)			99.614 (Pass)		

Sensitivity on $x^{(2)}_0$															
$N_e$	1			2			3			4			5		
Channel	R	G	B	R	G	B	R	G	B	R	G	B	R	G	B
Baboon	99.590	99.612	99.606	99.596	99.612	99.621	99.578	99.620	99.608	99.610	99.633	99.608	99.606	99.602	99.594
Flowers	99.605	99.591	99.609	99.622	99.610	99.619	99.595	99.636	99.628	99.610	99.610	99.611	99.606	99.607	99.606
Peppers	99.621	99.623	99.599	99.617	99.609	99.625	99.614	99.622	99.632	99.626	99.616	99.600	99.616	99.614	99.621
Goldhill	99.609	99.605	99.621	99.623	99.608	99.607	99.592	99.606	99.615	99.617	99.608	99.608	99.613	99.604	99.598
Black	99.588			99.613			99.612			99.619			99.616		
Boats	99.602			99.606			99.595			99.619			99.610		
Clock	99.559			99.599			99.657			99.643			99.590		
White	99.598			99.623			99.624			99.614			99.608		
Average	99.602 (Pass)			99.613 (Pass)			99.615 (Pass)			99.616 (Pass)			99.607 (Pass)		

Sensitivity on $x^{(1)}_0$															
$N_e$	1			2			3			4			5		
Channel	R	G	B	R	G	B	R	G	B	R	G	B	R	G	B
Baboon	99.608	99.624	99.592	99.600	99.611	99.612	99.605	99.633	99.618	99.635	99.605	99.607	99.604	99.620	99.610
Flowers	99.596	99.617	99.599	99.619	99.643	99.592	99.575	99.631	99.613	99.622	99.617	99.625	99.590	99.631	99.627
Peppers	99.623	99.607	99.616	99.603	99.614	99.625	99.624	99.622	99.599	99.617	99.620	99.621	99.619	99.621	99.599
Goldhill	99.592	99.619	99.625	99.615	99.617	99.593	99.608	99.608	99.610	99.625	99.614	99.589	99.606	99.605	99.607
Black	99.604			99.615			99.605			99.613			99.614		
Boats	99.611			99.598			99.608			99.610			99.607		
Clock	99.594			99.619			99.625			99.637			99.580		
White	99.628			99.600			99.610			99.632			99.609		
Average	99.610 (Pass)			99.611 (Pass)			99.612 (Pass)			99.618 (Pass)			99.609 (Pass)		

Sensitivity on $S_0$															
$N_e$	1			2			3			4			5		
Channel	R	G	B	R	G	B	R	G	B	R	G	B	R	G	B
Baboon	99.624	99.605	99.615	99.614	99.619	99.597	99.600	99.623	99.603	99.611	99.601	99.616	99.605	99.593	99.633
Flowers	99.588	99.609	99.599	99.613	99.606	99.592	99.603	99.609	99.639	99.620	99.612	99.611	99.614	99.599	99.606
Peppers	99.589	99.600	99.597	99.605	99.623	99.616	99.624	99.611	99.632	99.615	99.604	99.613	99.606	99.597	99.595
Goldhill	99.592	99.618	99.603	99.623	99.608	99.601	99.606	99.609	99.621	99.621	99.613	99.588	99.596	99.602	99.611
Black	99.620			99.615			99.621			99.610			99.620		
Boats	99.611			99.607			99.595			99.604			99.606		
Clock	99.632			99.606			99.657			99.600			99.611		
White	99.620			99.599			99.593			99.594			99.603		
Average	99.608 (Pass)			99.609 (Pass)			99.621 (Pass)			99.610 (Pass)			99.615 (Pass)

**Table 18:** Exemplar simulation for Structure 3 in Fig. 1c: Sensitivity of secret key by NPCR.

Sensitivity on $\gamma ^{(2)}_0$															
$N_e$	1			2			3			4			5		
Channel	R	G	B	R	G	B	R	G	B	R	G	B	R	G	B
Baboon	99.606	99.620	99.617	99.607	99.587	99.615	99.617	99.595	99.613	99.602	99.601	99.602	99.615	99.611	99.597
Flowers	99.606	99.603	99.610	99.610	99.604	99.587	99.610	99.617	99.586	99.622	99.599	99.601	99.597	99.603	99.610
Peppers	99.619	99.620	99.617	99.601	99.621	99.624	99.623	99.609	99.607	99.621	99.596	99.607	99.612	99.621	99.626
Goldhill	99.601	99.618	99.609	99.620	99.599	99.602	99.612	99.607	99.623	99.605	99.600	99.608	99.606	99.602	99.607
Black	99.613			99.619			99.612			99.592			99.614		
Boats	99.602			99.631			99.613			99.603			99.618		
Clock	99.635			99.661			99.611			99.599			99.622		
White	99.606			99.603			99.607			99.603			99.600		
Average	99.613 (Pass)			99.612 (Pass)			99.610 (Pass)			99.604 (Pass)			99.610 (Pass)		

Sensitivity on $\gamma ^{(1)}_0$															
$N_e$	1			2			3			4			5		
Channel	R	G	B	R	G	B	R	G	B	R	G	B	R	G	B
Baboon	99.625	99.592	99.625	99.608	99.602	99.607	99.590	99.639	99.610	99.604	99.598	99.627	99.614	99.593	99.615
Flowers	99.608	99.595	99.585	99.636	99.626	99.635	99.636	99.577	99.610	99.614	99.591	99.583	99.624	99.634	99.606
Peppers	99.618	99.617	99.622	99.599	99.611	99.624	99.631	99.607	99.615	99.609	99.617	99.621	99.622	99.617	99.598
Goldhill	99.602	99.616	99.615	99.608	99.604	99.631	99.605	99.619	99.614	99.606	99.601	99.596	99.617	99.595	99.612
Black	99.600			99.610			99.604			99.605			99.596		
Boats	99.603			99.616			99.588			99.611			99.615		
Clock	99.647			99.641			99.576			99.586			99.641		
White	99.607			99.610			99.604			99.598			99.618		
Average	99.611 (Pass)			99.617 (Pass)			99.608 (Pass)			99.604 (Pass)			99.614 (Pass)		

Sensitivity on $x^{(2)}_0$															
$N_e$	1			2			3			4			5		
Channel	R	G	B	R	G	B	R	G	B	R	G	B	R	G	B
Baboon	99.605	99.591	99.607	99.615	99.585	99.590	99.624	99.615	99.615	99.598	99.584	99.605	99.612	99.614	99.633
Flowers	99.584	99.632	99.593	99.616	99.608	99.627	99.613	99.605	99.606	99.601	99.615	99.604	99.598	99.626	99.619
Peppers	99.618	99.620	99.599	99.619	99.600	99.611	99.614	99.592	99.618	99.631	99.614	99.604	99.622	99.604	99.593
Goldhill	99.603	99.610	99.617	99.606	99.611	99.597	99.619	99.611	99.610	99.615	99.608	99.623	99.588	99.592	99.616
Black	99.625			99.617			99.605			99.607			99.607		
Boats	99.612			99.603			99.595			99.622			99.629		
Clock	99.576			99.635			99.606			99.612			99.617		
White	99.612			99.604			99.600			99.606			99.603		
Average	99.607 (Pass)			99.609 (Pass)			99.609 (Pass)			99.609 (Pass)			99.611 (Pass)		

Sensitivity on $x^{(1)}_0$															
$N_e$	1			2			3			4			5		
Channel	R	G	B	R	G	B	R	G	B	R	G	B	R	G	B
Baboon	99.606	99.591	99.628	99.605	99.605	99.602	99.594	99.611	99.611	99.624	99.602	99.598	99.610	99.605	99.598
Flowers	99.604	99.608	99.636	99.633	99.596	99.603	99.590	99.615	99.609	99.586	99.601	99.610	99.601	99.614	99.619
Peppers	99.615	99.599	99.609	99.620	99.617	99.619	99.621	99.600	99.591	99.601	99.621	99.623	99.607	99.608	99.619
Goldhill	99.617	99.609	99.614	99.619	99.612	99.609	99.619	99.626	99.605	99.612	99.628	99.592	99.610	99.596	99.612
Black	99.601			99.626			99.610			99.622			99.605		
Boats	99.612			99.584			99.621			99.606			99.613		
Clock	99.590			99.626			99.629			99.649			99.600		
White	99.610			99.609			99.606			99.596			99.615		
Average	99.609 (Pass)			99.612 (Pass)			99.610 (Pass)			99.611 (Pass)			99.608 (Pass)		

Sensitivity on $S_0$															
$N_e$	1			2			3			4			5		
Channel	R	G	B	R	G	B	R	G	B	R	G	B	R	G	B
Baboon	99.610	99.606	99.608	99.602	99.610	99.615	99.620	99.623	99.603	99.623	99.604	99.622	99.617	99.613	99.602
Flowers	99.599	99.609	99.588	99.602	99.599	99.602	99.613	99.622	99.628	99.617	99.607	99.608	99.570	99.622	99.592
Peppers	99.618	99.621	99.591	99.621	99.617	99.602	99.599	99.608	99.614	99.597	99.603	99.621	99.623	99.619	99.611
Goldhill	99.597	99.617	99.607	99.628	99.613	99.612	99.603	99.616	99.613	99.610	99.607	99.615	99.609	99.602	99.635
Black	99.618			99.618			99.605			99.590			99.604		
Boats	99.616			99.626			99.609			99.596			99.603		
Clock	99.602			99.657			99.594			99.574			99.580		
White	99.617			99.611			99.608			99.621			99.620		
Average	99.608 (Pass)			99.615 (Pass)			99.611 (Pass)			99.607 (Pass)			99.608 (Pass)

**Table 19:** Exemplar simulation for Structure 1 in Fig. 1a: Sensitivity of secret key by UACI.

Sensitivity on $\gamma ^{(2)}_0$															
$N_e$	1			2			3			4			5		
Channel	R	G	B	R	G	B	R	G	B	R	G	B	R	G	B
Baboon	33.378	33.502	33.427	33.487	33.428	33.368	33.427	33.487	33.476	33.395	33.451	33.473	33.454	33.498	33.452
Flowers	33.514	33.456	33.465	33.461	33.462	33.519	33.469	33.518	33.406	33.461	33.552	33.519	33.383	33.424	33.529
Peppers	33.440	33.455	33.478	33.506	33.540	33.499	33.410	33.454	33.351	33.450	33.421	33.432	33.444	33.421	33.500
Goldhill	33.449	33.475	33.422	33.460	33.439	33.473	33.437	33.512	33.468	33.420	33.435	33.452	33.499	33.440	33.470
Black	33.492			33.457			33.446			33.532			33.511		
Boats	33.443			33.485			33.510			33.480			33.460		
Clock	33.580			33.568			33.447			33.474			33.360		
White	33.490			33.401			33.525			33.505			33.468		
Average	33.467 (Pass)			33.472 (Pass)			33.459 (Pass)			33.466 (Pass)			33.457 (Pass)		

Sensitivity on $\gamma ^{(1)}_0$															
$N_e$	1			2			3			4			5		
Channel	R	G	B	R	G	B	R	G	B	R	G	B	R	G	B
Baboon	33.485	33.417	33.565	33.533	33.441	33.486	33.526	33.505	33.466	33.395	33.477	33.537	33.466	33.353	33.495
Flowers	33.419	33.503	33.422	33.531	33.471	33.470	33.458	33.539	33.435	33.474	33.491	33.509	33.428	33.606	33.337
Peppers	33.467	33.354	33.532	33.431	33.513	33.420	33.415	33.499	33.459	33.464	33.529	33.489	33.541	33.430	33.424
Goldhill	33.492	33.448	33.422	33.467	33.507	33.532	33.495	33.448	33.453	33.459	33.436	33.466	33.450	33.487	33.424
Black	33.493			33.477			33.463			33.451			33.441		
Boats	33.490			33.494			33.483			33.489			33.465		
Clock	33.643			33.481			33.398			33.564			33.425		
White	33.476			33.454			33.438			33.446			33.469		
Average	33.477 (Pass)			33.482 (Pass)			33.468 (Pass)			33.480 (Pass)			33.453 (Pass)		

Sensitivity on $x^{(2)}_0$															
$N_e$	1			2			3			4			5		
Channel	R	G	B	R	G	B	R	G	B	R	G	B	R	G	B
Baboon	33.443	33.405	33.436	33.426	33.391	33.423	33.515	33.496	33.520	33.519	33.483	33.371	33.411	33.475	33.512
Flowers	33.462	33.555	33.549	33.460	33.421	33.405	33.503	33.488	33.467	33.445	33.475	33.531	33.476	33.531	33.429
Peppers	33.495	33.531	33.488	33.387	33.549	33.455	33.448	33.511	33.520	33.459	33.470	33.467	33.433	33.424	33.445
Goldhill	33.469	33.463	33.445	33.453	33.448	33.392	33.497	33.512	33.444	33.489	33.504	33.450	33.420	33.437	33.449
Black	33.461			33.492			33.464			33.489			33.465		
Boats	33.458			33.396			33.449			33.472			33.465		
Clock	33.515			33.541			33.386			33.385			33.519		
White	33.484			33.485			33.468			33.449			33.449		
Average	33.479 (Pass)			33.445 (Pass)			33.481 (Pass)			33.466 (Pass)			33.459 (Pass)		

Sensitivity on $x^{(1)}_0$															
$N_e$	1			2			3			4			5		
Channel	R	G	B	R	G	B	R	G	B	R	G	B	R	G	B
Baboon	33.520	33.447	33.503	33.472	33.469	33.395	33.428	33.500	33.526	33.484	33.541	33.491	33.430	33.395	33.450
Flowers	33.429	33.483	33.398	33.396	33.450	33.453	33.456	33.415	33.544	33.583	33.418	33.517	33.411	33.405	33.288
Peppers	33.469	33.499	33.440	33.459	33.438	33.487	33.518	33.379	33.500	33.400	33.529	33.449	33.488	33.411	33.445
Goldhill	33.517	33.447	33.444	33.455	33.476	33.489	33.423	33.452	33.406	33.420	33.476	33.451	33.414	33.461	33.440
Black	33.516			33.490			33.441			33.485			33.532		
Boats	33.454			33.390			33.505			33.530			33.457		
Clock	33.532			33.471			33.494			33.548			33.258		
White	33.496			33.492			33.416			33.451			33.532		
Average	33.475 (Pass)			33.455 (Pass)			33.463 (Pass)			33.486 (Pass)			33.426 (Pass)		

Sensitivity on $S_0$															
$N_e$	1			2			3			4			5		
Channel	R	G	B	R	G	B	R	G	B	R	G	B	R	G	B
Baboon	33.466	33.523	33.434	33.520	33.432	33.502	33.528	33.487	33.525	33.458	33.445	33.446	33.498	33.473	33.435
Flowers	33.450	33.456	33.519	33.409	33.457	33.484	33.462	33.487	33.525	33.478	33.388	33.551	33.441	33.579	33.440
Peppers	33.489	33.470	33.366	33.439	33.433	33.412	33.435	33.420	33.449	33.513	33.499	33.417	33.458	33.537	33.540
Goldhill	33.527	33.423	33.525	33.469	33.419	33.474	33.501	33.483	33.474	33.422	33.478	33.442	33.478	33.453	33.512
Black	33.426			33.551			33.431			33.446			33.526		
Boats	33.497			33.449			33.436			33.465			33.446		
Clock	33.501			33.633			33.383			33.573			33.471		
White	33.494			33.492			33.518			33.496			33.521		
Average	33.447 (Pass)			33.448 (Pass)			33.483 (Pass)			33.466 (Pass)			33.460 (Pass)

**Table 20:** Exemplar simulation for Structure 2 in Fig. 1b: Sensitivity of secret key by UACI.

Sensitivity on $\gamma ^{(2)}_0$															
$N_e$	1			2			3			4			5		
Channel	R	G	B	R	G	B	R	G	B	R	G	B	R	G	B
Baboon	33.375	33.499	33.403	33.513	33.421	33.462	33.549	33.462	33.426	33.391	33.476	33.471	33.422	33.364	33.465
Flowers	33.432	33.414	33.429	33.483	33.447	33.495	33.475	33.578	33.567	33.459	33.535	33.415	33.369	33.431	33.440
Peppers	33.419	33.420	33.511	33.547	33.509	33.455	33.389	33.531	33.460	33.477	33.466	33.507	33.478	33.445	33.446
Goldhill	33.432	33.470	33.445	33.436	33.455	33.453	33.432	33.444	33.471	33.463	33.424	33.498	33.454	33.495	33.495
Black	33.510			33.416			33.531			33.535			33.492		
Boats	33.442			33.504			33.451			33.480			33.409		
Clock	33.572			33.403			33.456			33.513			33.497		
White	33.490			33.396			33.441			33.511			33.513		
Average	33.454 (Pass)			33.462 (Pass)			33.479 (Pass)			33.476 (Pass)			33.451 (Pass)		

Sensitivity on $\gamma ^{(1)}_0$															
$N_e$	1			2			3			4			5		
Channel	R	G	B	R	G	B	R	G	B	R	G	B	R	G	B
Baboon	33.420	33.398	33.506	33.502	33.445	33.379	33.566	33.486	33.497	33.416	33.438	33.554	33.489	33.536	33.472
Flowers	33.500	33.466	33.451	33.472	33.515	33.421	33.418	33.445	33.435	33.485	33.520	33.496	33.436	33.455	33.479
Peppers	33.413	33.357	33.466	33.460	33.423	33.519	33.406	33.398	33.429	33.457	33.429	33.492	33.489	33.527	33.440
Goldhill	33.461	33.442	33.435	33.468	33.363	33.499	33.464	33.406	33.492	33.495	33.466	33.429	33.380	33.460	33.500
Black	33.545			33.503			33.541			33.474			33.521		
Boats	33.417			33.486			33.456			33.436			33.419		
Clock	33.418			33.328			33.418			33.281			33.537		
White	33.496			33.444			33.481			33.458			33.437		
Average	33.449 (Pass)			33.452 (Pass)			33.459 (Pass)			33.458 (Pass)			33.474 (Pass)		

Sensitivity on $x^{(2)}_0$															
$N_e$	1			2			3			4			5		
Channel	R	G	B	R	G	B	R	G	B	R	G	B	R	G	B
Baboon	33.455	33.417	33.542	33.451	33.407	33.397	33.431	33.423	33.399	33.501	33.520	33.486	33.412	33.492	33.447
Flowers	33.398	33.522	33.477	33.501	33.491	33.475	33.513	33.402	33.525	33.451	33.387	33.398	33.446	33.481	33.469
Peppers	33.424	33.460	33.489	33.406	33.418	33.492	33.466	33.543	33.432	33.551	33.428	33.564	33.460	33.470	33.463
Goldhill	33.471	33.450	33.490	33.452	33.443	33.406	33.467	33.436	33.448	33.479	33.435	33.413	33.483	33.485	33.517
Black	33.460			33.469			33.484			33.510			33.409		
Boats	33.446			33.461			33.451			33.472			33.505		
Clock	33.570			33.325			33.557			33.473			33.364		
White	33.425			33.438			33.530			33.503			33.450		
Average	33.469 (Pass)			33.440 (Pass)			33.469 (Pass)			33.473 (Pass)			33.460 (Pass)		

Sensitivity on $S_0$															
$N_e$	1			2			3			4			5		
Channel	R	G	B	R	G	B	R	G	B	R	G	B	R	G	B
Baboon	33.521	33.452	33.544	33.473	33.478	33.452	33.518	33.519	33.416	33.527	33.499	33.463	33.491	33.498	33.395
Flowers	33.468	33.430	33.471	33.483	33.528	33.482	33.421	33.434	33.533	33.491	33.437	33.475	33.439	33.388	33.569
Peppers	33.499	33.439	33.459	33.432	33.547	33.555	33.429	33.443	33.394	33.401	33.425	33.433	33.321	33.468	33.412
Goldhill	33.448	33.523	33.439	33.474	33.494	33.429	33.499	33.413	33.469	33.440	33.478	33.406	33.487	33.473	33.497
Black	33.452			33.479			33.520			33.485			33.519		
Boats	33.481			33.449			33.500			33.406			33.399		
Clock	33.502			33.628			33.463			33.514			33.451		
White	33.458			33.500			33.517			33.516			33.520		
Average	33.470 (Pass)			33.474 (Pass)			33.453 (Pass)			33.477 (Pass)			33.490 (Pass)

**Table 21:** Exemplar simulation for Structure 3 in Fig. 1c: Sensitivity of secret key by UACI.

Sensitivity on $\gamma ^{(2)}_0$															
$N_e$	1			2			3			4			5		
Channel	R	G	B	R	G	B	R	G	B	R	G	B	R	G	B
Baboon	33.378	33.502	33.427	33.487	33.428	33.368	33.427	33.487	33.476	33.395	33.451	33.473	33.454	33.498	33.452
Flowers	33.514	33.456	33.465	33.461	33.462	33.519	33.469	33.518	33.406	33.461	33.552	33.519	33.383	33.424	33.529
Peppers	33.441	33.497	33.463	33.527	33.551	33.447	33.420	33.439	33.401	33.417	33.434	33.407	33.424	33.433	33.547
Goldhill	33.449	33.475	33.422	33.460	33.439	33.473	33.437	33.512	33.468	33.420	33.435	33.452	33.499	33.440	33.470
Black	33.492			33.457			33.446			33.532			33.511		
Boats	33.443			33.485			33.510			33.480			33.460		
Clock	33.580			33.568			33.447			33.474			33.360		
White	33.490			33.401			33.525			33.505			33.468		
Average	33.468 (Pass)			33.471 (Pass)			33.462 (Pass)			33.463 (Pass)			33.460 (Pass)		

Sensitivity on $\gamma ^{(1)}_0$															
$N_e$	1			2			3			4			5		
Channel	R	G	B	R	G	B	R	G	B	R	G	B	R	G	B
Baboon	33.485	33.417	33.565	33.533	33.441	33.486	33.526	33.505	33.466	33.395	33.477	33.537	33.466	33.353	33.495
Flowers	33.419	33.503	33.422	33.531	33.471	33.470	33.458	33.539	33.435	33.474	33.491	33.509	33.428	33.606	33.337
Peppers	33.469	33.441	33.507	33.430	33.483	33.420	33.431	33.514	33.452	33.467	33.527	33.488	33.526	33.417	33.430
Goldhill	33.492	33.448	33.422	33.467	33.507	33.532	33.495	33.448	33.453	33.459	33.436	33.466	33.450	33.487	33.424
Black	33.493			33.477			33.463			33.451			33.441		
Boats	33.490			33.494			33.483			33.489			33.465		
Clock	33.643			33.481			33.398			33.564			33.425		
White	33.476			33.454			33.438			33.446			33.469		
Average	33.481 (Pass)			33.480 (Pass)			33.469 (Pass)			33.480 (Pass)			33.451 (Pass)		

Sensitivity on $x^{(2)}_0$															
$N_e$	1			2			3			4			5		
Channel	R	G	B	R	G	B	R	G	B	R	G	B	R	G	B
Baboon	33.443	33.405	33.436	33.426	33.391	33.423	33.515	33.496	33.520	33.519	33.483	33.371	33.411	33.475	33.512
Flowers	33.462	33.555	33.549	33.460	33.421	33.405	33.503	33.488	33.467	33.445	33.475	33.531	33.476	33.531	33.429
Peppers	33.525	33.531	33.469	33.411	33.543	33.459	33.434	33.500	33.471	33.436	33.448	33.455	33.409	33.424	33.432
Goldhill	33.469	33.463	33.445	33.453	33.448	33.392	33.497	33.512	33.444	33.489	33.504	33.450	33.420	33.437	33.449
Black	33.461			33.492			33.464			33.489			33.465		
Boats	33.458			33.396			33.449			33.472			33.465		
Clock	33.515			33.541			33.386			33.385			33.519		
White	33.484			33.485			33.468			33.449			33.449		
Average	33.479 (Pass)			33.447 (Pass)			33.476 (Pass)			33.463 (Pass)			33.456 (Pass)		

Sensitivity on $x^{(1)}_0$															
$N_e$	1			2			3			4			5		
Channel	R	G	B	R	G	B	R	G	B	R	G	B	R	G	B
Baboon	33.520	33.447	33.503	33.472	33.469	33.395	33.428	33.500	33.526	33.484	33.541	33.491	33.430	33.395	33.450
Flowers	33.429	33.483	33.398	33.396	33.450	33.453	33.456	33.415	33.544	33.583	33.418	33.517	33.411	33.405	33.288
Peppers	33.469	33.517	33.442	33.436	33.418	33.465	33.517	33.393	33.500	33.425	33.439	33.441	33.469	33.427	33.435
Goldhill	33.517	33.447	33.444	33.455	33.476	33.489	33.423	33.452	33.406	33.420	33.476	33.451	33.414	33.461	33.440
Black	33.516			33.490			33.441			33.485			33.532		
Boats	33.454			33.390			33.505			33.530			33.457		
Clock	33.532			33.471			33.494			33.548			33.258		
White	33.496			33.492			33.416			33.451			33.532		
Average	33.476 (Pass)			33.451 (Pass)			33.464 (Pass)			33.481 (Pass)			33.425 (Pass)		

Sensitivity on $S_0$															
$N_e$	1			2			3			4			5		
Channel	R	G	B	R	G	B	R	G	B	R	G	B	R	G	B
Baboon	33.466	33.523	33.434	33.520	33.432	33.502	33.528	33.487	33.525	33.458	33.445	33.446	33.498	33.473	33.435
Flowers	33.450	33.456	33.519	33.409	33.457	33.484	33.462	33.487	33.525	33.478	33.388	33.551	33.441	33.579	33.440
Peppers	33.469	33.457	33.371	33.435	33.417	33.434	33.422	33.439	33.441	33.520	33.514	33.426	33.438	33.529	33.541
Goldhill	33.527	33.423	33.525	33.469	33.419	33.474	33.501	33.483	33.474	33.422	33.478	33.442	33.478	33.453	33.512
Black	33.426			33.551			33.431			33.446			33.526		
Boats	33.497			33.449			33.436			33.465			33.446		
Clock	33.501			33.633			33.383			33.573			33.471		
White	33.494			33.492			33.518			33.496			33.433		
Average	33.471 (Pass)			33.474 (Pass)			33.471 (Pass)			33.472 (Pass)			33.481 (Pass)		

### Comparison to the simulation results of some algorithms of chaos-based MIE

Table 22 shows the comparison between the exemplar simulation using the proposed structures and some existing MIE algorithms based on chaos. Here, comparisons are made only on the statistical properties of ciphertexts and the security properties of algorithms, regardless of structural configuration, parallelism, techniques, and image-content dependency. It is clear that the statistical properties of the ciphertexts in this simulation, i.e. information entropy and correlation coefficients, are comparable to those of existing algorithms. Although the key space of the exemplar designs ($2^{486}$) is moderate compared to that of other algorithms, it is large enough to resist the brute-force attack using a modern computer. The randomness of ciphertexts and sensitivity of secret key in this simulation are measured with pass by means of the chi-square test and of NCPR and UAIC, while some other algorithms are not quantitatively reported.

**Table 22:** Comparison with existing algorithms of chaos-based MIE.

Algorithms	Statistical analysis					Security analysis		
	IE	Corr. coeff. (worse cases on average)			$\chi ^2$	Key Space	Sensitivity of secr. key	
		Horizontal	Vertical	Diagonal			by NCPR	by UACI
Structure 1	7.9992	0.0001	-0.0003	-0.0004	Pass	$2^{486}$	Pass	Pass
Structure 2	7.9992	0.0003	0.0002	-0.0003	Pass	$2^{486}$	Pass	Pass
Structure 3	7.9993	-0.0001	0.0003	0.0001	Pass	$2^{486}$	Pass	Pass
Karawia^23^	7.9989	0.0002	0.0008	0.0008	-	$10^{210}\approx 1.5209 \times 2^{697}$	Pass	Pass
Zarebnia^25^	7.9995	-0.0036	0.00262	0.001239	-	$10^{195} \approx 1.7124\times 2^{647}$	-	-
Patro^49^	7.9993	-0.0003	-0.0001	-0.0002	Pass	$1.2446\times 2^{327}$	Pass	Pass
Zhang^6^	7.9993	-0.0003	0.0003	-0.0002	-	$10^{56}\approx 1.0195 \times 2^{186}$	-	-
Malik^24^	7.9993	0.0000	0.0008	0.0005	-	$10^{165} \approx 1.3653 \times 2^{548}$	-	-
Patro^26^	7.9993	-0.0006	-0.0001	-0.0020	-	$1.964 \times 2^{426}$	Pass	Pass
Patro^27^	7.9974	-0.0012	-0.0015	0.0005	-	$1.964 \times 2^{426}$	Pass	Pass
Hoang^28^	7.9972	0.0031	0.0018	0.0002	Pass	$2^{332}$	Pass	Pass
Hoang^29^	7.9971	-0.0019	0.0018	-0.0010	Pass	$2^{230}$ (Logistic map) and $2^{230}$ (Standard map)	Pass	Pass
Sahasrabuddhe^30^	7.9999	-0.0036	0.0016	-0.0058	-	$9.4599\times 10^{76}\approx 1.634\times 2^{255}$	-	-
Jahangir^31^	7.9979	-0.0002	0.0000	-0.0036	-	-	-	-
Zhou^33^	7.9999	-0.0017	-0.0006	0.0007	-	$10^{15\times 8} \approx 1.5491\times 2^{398}$	-	-
Tanveer^34^	7.9963	0.0016	-0.0056	0.0015	-	$10^{105}\approx 2^{348}$	-	-
Liu^39^	7.9897	-0.0119	-0.0087	-0.0045	-	$10^{15}\times 16\approx 1.1997\times 2^{797}$	-	-
Chen^40^	7.9973	-0.0023	0.0024	0.0011	-	$10^{103} \times 2^{108}\approx 1.1162 \times 2^{450}$	Pass	Pass
Qiu^42^	7.9991	0.0008	0.0007	-0.0003	-	$2^{512}$	-	-
Joshi^46^	7.9959	-0.0223	-0.0056	-0.0274	-	$10^{75} \approx 2^{250}$	-	-
Tang^47^	-	0.0705	-0.1504	-0.0491	-	$8 \times 10^{63} \approx 1.2155\times 2^{212}$	-	-
Kaur^35^	7.9952	0.0013	0.0020	0.0042	-	$10^{247} \approx 1.4302 \times 2^{820}$	-	-
Gao^50^	7.9993	0.0021	0.0029	0.0023	Pass	$10^{249} \approx 2^{827}$	Pass	Pass
Haq^7^	7.9994	0.0072	-0.0002	0.0006	-	$2^{245}$	-	-
Zhang^36^	7.9992	0.0018	-0.0021	0.0018	-	$10^{135} \times (8k)! \approx 1.3712\times 2^{744}$ (8 images)	-	-
Zhang^32^	7.9936	-	-	-	-	-	-	-

The simulation shows by means of the exemplar ciphers that the proposed structures are effective for practical applications. Next, the hardware for Structure 1 is designed, simulated, verified, and implemented on the FPGA platform to confirm the feasibility of the proposed designs in hardware applications.

## Digital hardware design

In this section, the hardware for MIE Structure 1 as illustrated in Fig. 1a is designed and implemented on the Intel FPGA device. The reason for choosing the FPGA platform is that the pixels of an individual image are permuted in the range of the image. The GPU only outperforms the FPGA if an image is processed in separate portions of pixels. In addition, the pipeline is implemented in FPGA to parallelize the encryption process for every individual image.

The hardware design uses the value of parameters for in the simulation mentioned above. The results on resource utilization, throughput, and hardware performance will also be evaluated and discussed. The hardware architecture of decryption is analogous to that of encryption, but the difference is only the reverse order of pixel processing compared to that of encryption.

**Fig. 9:**
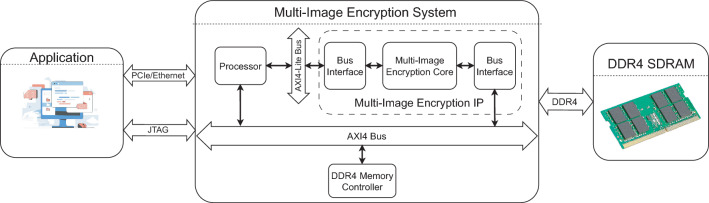
The hardware architecture.

### Hardware architecture of MIE

Figure 9 shows the MIE system architecture that incorporates MIE hardware as a core component, which is named MIE Intellectual Property (MIE-IP). The MIE-IP receives instructions from the processor through the AXI4-Lite communication interface to manage control information such as parameter setting for the encryption system, encryption requests, and read/write requests of data. The DDR4 SDRAM module is used to accommodate the image data for encryption or decryption. Both the processor and MIE-IP interface with the Memory Controller to access data in the DDR4 SDRAM. Furthermore, the application connects with the MIE system through high-speed data communication protocols such as PCIe or Ethernet. The operation of MIE System can be tracked through the JTAG communication interface.

### Operation of digital hardware

**Fig. 10:**
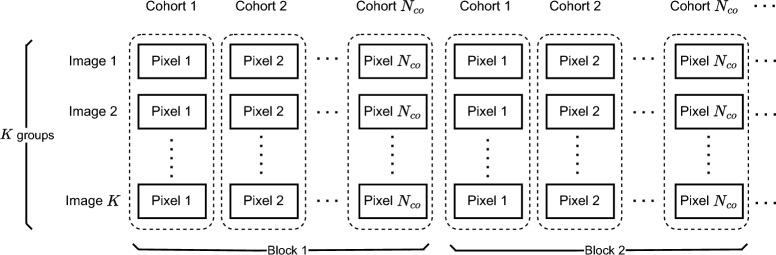
Blocks of pixels.

The MIE-IP hardware performs the encryption function described above. The execution time is balanced to have optimal speed for the entire system. So, the MIE system performs on blocks of $K\times N_{co}$ pixels in the internal memory, SRAM, of the FPGA chip. A block of pixels is illustrated in Fig. 10. A cohort of *K* pixels, each from an individual image, is encrypted at the same time. The encryption is repeated block by block until all images are encrypted. Encryption of a block is called an iteration. In each iteration, the hardware performs reading a block of data from the DDR4 SDRAM into the SRAM, encrypting blocks’ pixels, and writing encrypted pixels from the SRAM into the DDR4 SDRAM.

#### Memory reads/write

**Fig. 11:**
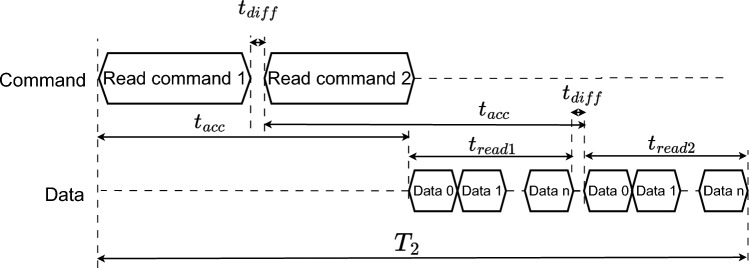
Timing diagram of the scheme of two consecutive read commands with the DDR4 SDRAM^76^.

The scheme of consecutive read/write commands is used for reading/writing blocks of pixels between the SRAM and the DDR4 SDRAM. As an example for two consecutive read commands, the detail of the time is shown in Fig. 11. Specifically, the duration is $T_2 = t_{acc} + t_{read1}+ t_{read2}+ t_{diff}$ with $t_{diff} \ll t_{acc}$ and $t_{diff} \ll t_{read}$; where $t_{acc}$ is the time access of the read commands; $t_{read}$ is the time for all data to be read, and $t_{diff}$ is the delay between two read commands or between two groups of data from consecutive read commands. Multiple consecutive read commands can be performed to save time. This is also applicable to write commands.

#### Operation phases of digital hardware

**Fig. 12:**
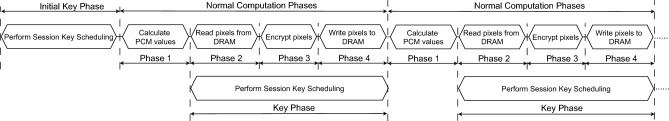
Operation phases of MIE-IP.

In this work, the allocation of time for the operating phases of the hardware is illustrated in Fig. 12. The encryption process is divided into multiple iterations and each iteration encrypts a block of pixels. An iteration consists of a series of phases, that is, Initial Key Phase and Normal Computation Phases, as shown in Fig. 12.

At the beginning of MIE, the SKS performs Initial Key Phase, and then the PCM iterates for the first value in Phase 1. Blocks of pixels are read from the DDR4 SDRAM into the SRAM, encrypted in SDRAM, and written back to the DDR4 SDRAM in Phases 2, 3, and 4. In order to optimize computation time, Session Key Scheduling, called Key Phase, for the next block is performed concurrently with Phases 2, 3, and 4 of the current block.

### MIE-IP architecture

**Fig. 13:**
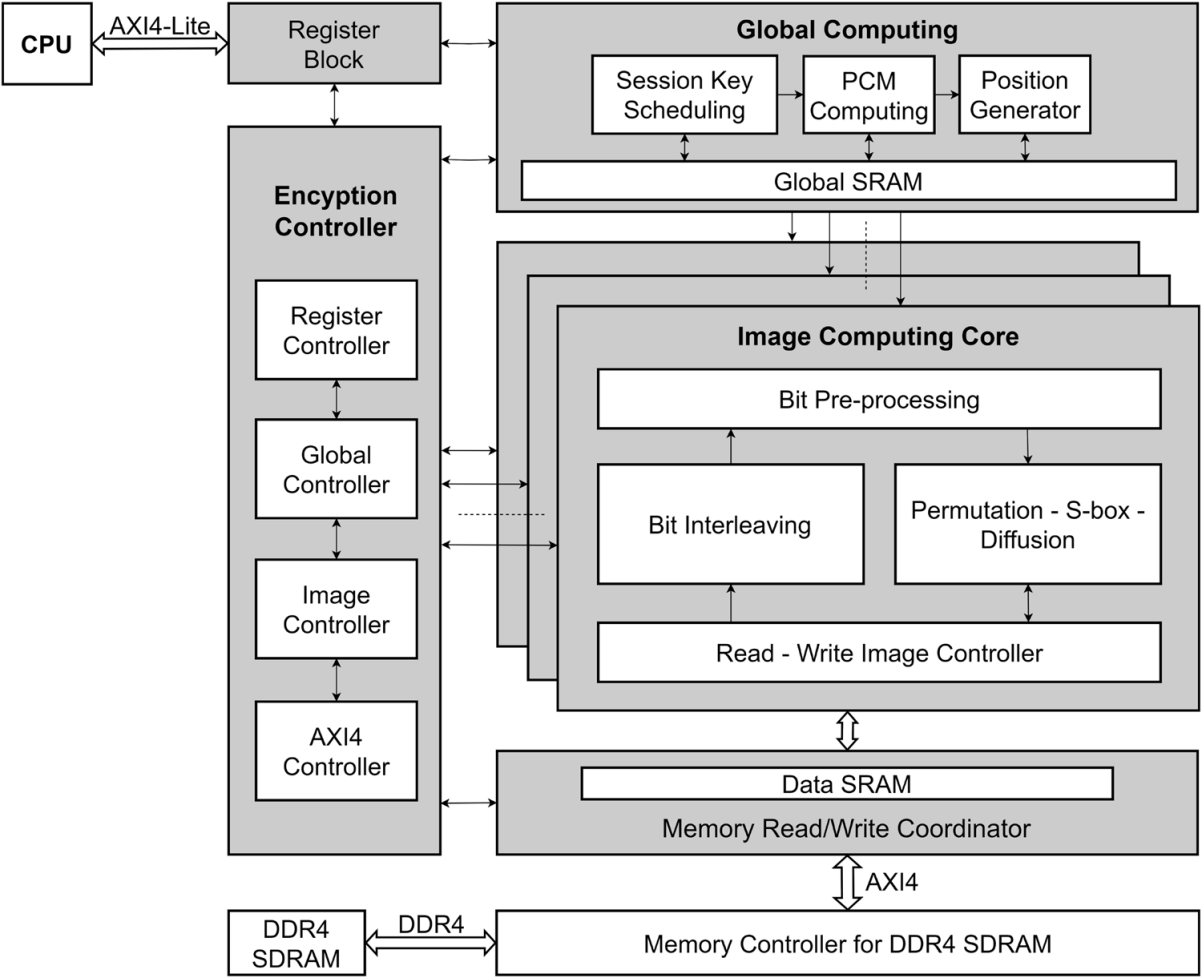
The abstract architecture of the MIE-IP.

The abstract architecture of MIE-IP is shown in Fig. 13. The design consists of five key components, as detailed in the following.

#### Register block

Register Block (RB) is a collection of registers that are used to store parameters, operational information, and system status information. Processor (CPU) writes control information to and reads status information from the RB via the AXI4-Lite communication interface.

#### Global computing

Global Computing (GC) consists of three main components: Session Key Scheduling, PCM Computing, and Position Generator.

Session Key Scheduling (SKS) works as an LFSR-based keystream generator. The number of states for the LFSR in the SKS is calculated based on Eq. (12), i.e., $N= 32$ states as in the simulation. However, the number of bits required to encrypt a cohort of *K* pixels is equal to the length of a frame given in Fig. 3, i.e., $L_E+K*L_{sk}= 608$ bits in this exemplar design. With an LFSR of 32 states, it requires 608 clock cycles to obtain 608 bits. To reduce the time delay in generating a sequence of 608 bits, this design implements the pipeline of 19 parallel-running LFSRs, and each LSFR is 32 bits in length. The seed $S_0$ of 32 bits is modified to become 19 seeds for 19 LFSRs. The bit sequences in the outputs of 19 LFSRs are merged to become 608 bits. In other words, the sequence of 608 bits is generated in only 32 clock cycles.

**Fig. 14:**
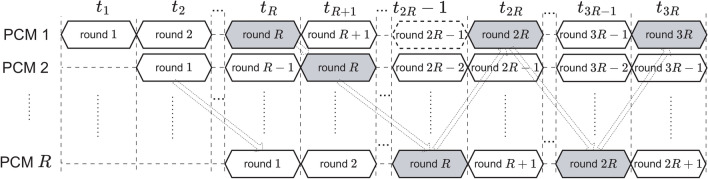
Timing diagram of PCM pipelining computing.

PCM Computing (PC) performs *R* chaotic iterations to get a value vector of $X_R$. So, a block of $N_{co}$ cohorts requires $N_{co}$ values of $X_R$. In order to optimize the speed of the hardware, the pipeline technique is applied to the hardware design for the PC, in which *R* PCMs are implemented and run in parallel using the same clock signal. However, such the *R* PCMs are put together in chain to reduce the computation time that the output of a PCM is the input of another. Therefore, instead of $R*N_{co}$ clock cycles to obtain $N_{co}$ values of $X_R$, it requires only $R + N_{co}-1$ clock cycles as Fig. 14 illustrates the timing diagram of PCM Computing, where values of $X_R$ are obtained at $t_R$ in different PCMs. In this exemplar design, $N_{co}=8$ and $R=5$ are chosen, so it only requires 13 clock cycles, instead of 39 clock cycles.

Position Generator performs the computation using Eqs. (13) and (14), and is implemented on hardware using only combinational logic gates, so it does not require any clock cycle in the timing diagram of the system.

#### Image computing core

Each Image Computing Core (ICC) is responsible for encrypting pixels of an individual image. The more ICCs integrated into the system, the greater the number of images that can be encrypted simultaneously.

Each ICC composed of four primary components, i.e. Bit Pre-processing (BPP), Bit Interleaving (BI), Permutation-S-box-Diffusion (PSD), and Read-Write Image Controller (RWIC). The specific roles of BPP, BI, and PSD are detailed above. The RWIC component oversees the reading and writing processes of blocks of pixels from the Memory Read/Write Coordinator. The ICCs perform its computations in parallel, each ICC operates independently without interference from others.

#### Memory read/write coordinator

**Fig. 15:**
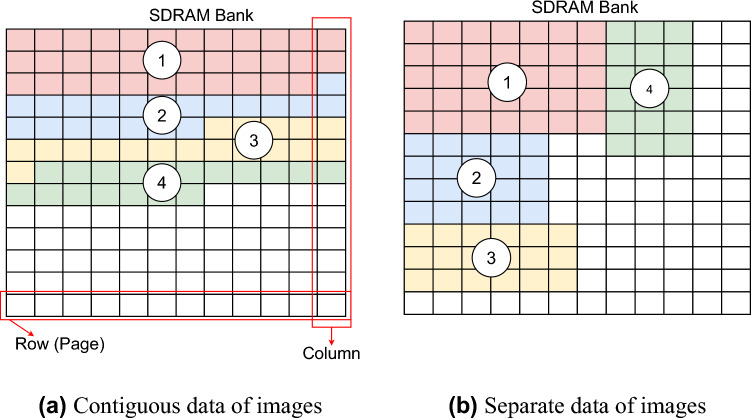
The method of organizing image data in the DDR4 SDRAM for images $\textcircled {1}$, $\textcircled {2}$, $\textcircled {3}$, and $\textcircled {4}$.

Memory Read/Write Coordinator (MRWC) is responsible for coordinating data transfer between the Memory Controller via the AXI4 interface and the ICCs. The MRWC uses the SRAM on the FPGA chip to temporarily store image data retrieved from the DDR4 SDRAM in Phase 2 and to temporarily hold encrypted image data before being written to the DDR4 SDRAM by MIE-IP. In addition, this block also performs the crucial function of converting pixel coordinates into corresponding storage addresses in the DDR4 SDRAM, and vice versa. There are two specific data organization methods as shown in Fig. 15, where pixels with the same highlighted color correspond to the same image.

The first method in Fig. 15a organizes the images in a continuous way. The second method in Fig. 15b allocates pixels of the same image to a separate memory region where pixels of the same image are non-adjacent addresses in the DDR4 SDRAM. In fact, the first method provides better optimization for memory space compared to the second. However, the first method creates complexity in the coordinate-address transformation process, i.e., the conversion of pixel coordinates into storage addresses in the DDR4 SDRAM. Consequently, the computational complexity of the first method of data organization reduces the overall encryption speed. The second method requires less hardware resources, compared to that in the first one, for the coordinate-address transformation. Since the amount of image data is relatively modest in comparison to the space of the DDR4 SDRAM, the second method is chosen to optimize the speed of the hardware.

#### Encryption controller

Encryption Controller (EC) oversees the entire operation of Global Computing, Image Computing Cores, and Memory Read/Write Coordinator. Specifically, the EC consists of subsystems with different responsibilities, that is, the Register Controller manages the read and write operations of Register Block; Global Controller coordinates the functions of Global Computing; Image Controller directs the activities of all Image Computing Cores; and the AXI4 Controller generates the control signals for the Memory Read/Write Coordinator.

**Fig. 16:**
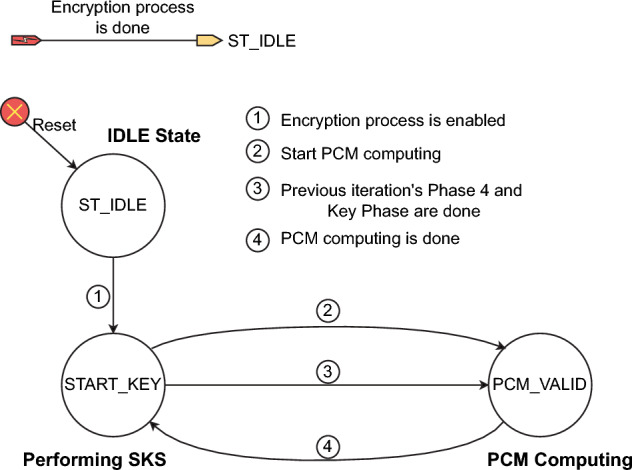
The FSM of Global Controller for a single cohort of pixels.

**Fig. 17:**
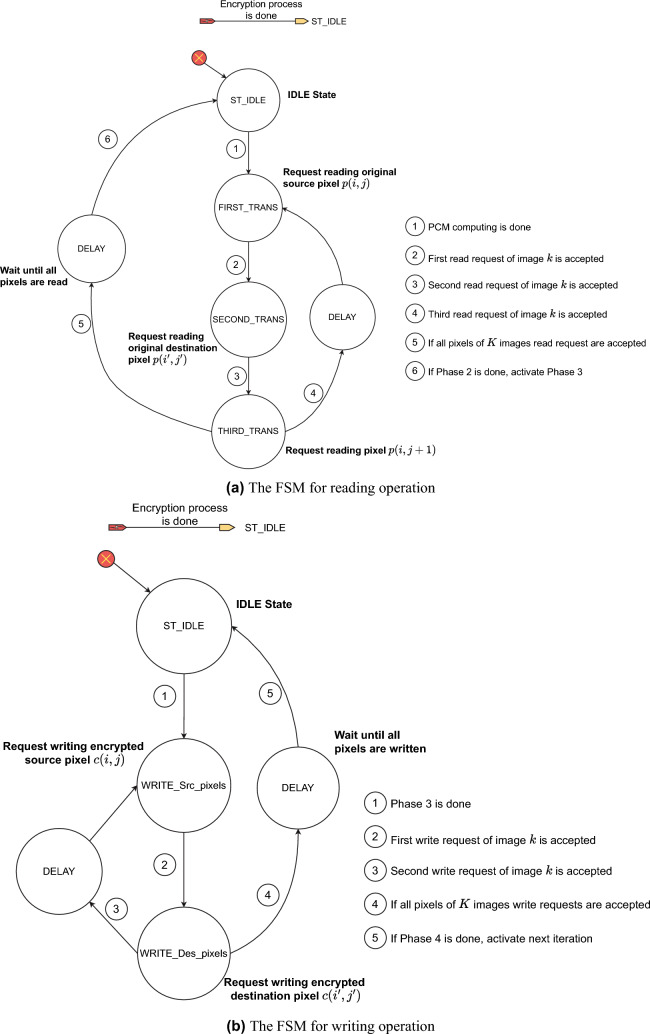
The FSMs of AXI4 Controller for reading and writing operations between the SRAM and the DDR4 SDRAM.

Global Controller operates on the basis of a finite state machine (FSM) as depicted in Fig. 16. The AXI4 controller consists of two FSMs, as illustrated in Fig. 17 to manage the reading and writing processes of MIE-IP using the AXI4 protocol.

### FPGA implementation

**Fig. 18:**
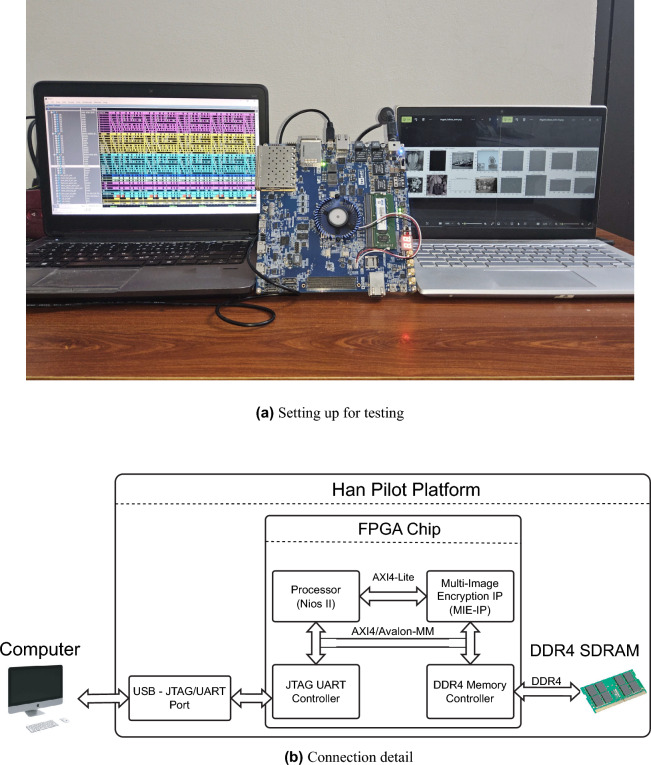
Testing model for the system.

The MIE system in Fig. 9 is implemented on the Han Pilot Platform and tested as shown in Fig. 18a to confirm the operation of the proposed design. The Han Pilot Platform is a development kit based on the Intel Arria 10 SoC FPGA from Intel-Altera. In this work, the code is written in SystemVerilog, then synthesized and implemented using Quartus Prime 21.1 Standard Edition. Functionality testing and performance evaluation are conducted for the implementation.

#### FPGA-based system architecture

Figure 18b shows the details of the MIE system on the Han Pilot Platform for testing; where the Nios II CPU is used for general control and the JTAG-UART is utilized for console communication.

#### Hardware metrics

The encryption and decryption processes of the FPGA-based system are simulated using Questa *-Intel® FPGA Edition software. After verifying the MIE-IP through functional simulation, the hardware metrics are assessed to demonstrate the utilization of the design resources, including the number of adaptive logic modules (ALM), digital signal processing blocks (DSP), registers, and block memory bits. The performance of the design is evaluated, including speed (maximum frequency, throughput), efficiency (throughput-to-ALM ratio), and power consumption.

- The throughput: Here, the throughput is computed for the maximum image data that goes through the hardware during the encryption time. It depends on, at maximum, the number of images that are encrypted at the same times, the size of the images, and the depth of the pixels as
  21 $$\begin{aligned} Throughput=\frac{Q_{max} * K_{max}*M_{max}*N_{max}}{T_{total}} ~~\text {(Mbps)}, \end{aligned}$$
  where $T_{total}$ is the encryption time for all data by only the ICCs. $T_{total}$ is counted by $T_{total}=\frac{N_{iterations}*T_{N_{co}}*N_{co}}{f_{max}}$, in which $f_{max}$ is the maximum frequency of the MIE-IP core; $N_{iterations}$ is the number of iterations needed to encrypt all pixels of the largest image, $N_{iterations}=\lceil \frac{M_{max}*N_{max}}{N_{co}}\rceil$; and $T_{N_{co}}$ is the number of clock cycles to encrypt plain pixels $p(i_k,j_k)$ and $p(i'_k,j'_k)$ for $k=1..K$ as
  22 $$\begin{aligned} T_{N_{co}}=T_{SDRAM\_to\_SRAM}+T_{SRAM\_to\_ICCs}+T_{ICCs\_to\_SRAM}+T_{SRAM\_to\_SDRAM}, \end{aligned}$$
  where $T_{SDRAM\_to\_SRAM}$ takes six clock cycles to write $p(i_k,j_k)$, $p(i'_k,j'_k)$, $c(i_k,j_k-1)$, $c(i'_k,j'_k-1)$, $p(i_k,j_k-1)$, $p(i'_k,j'_k-1)$ for $k=1..K$ from the DDR4 SDRAM to the SRAM; $T_{SRAM\_to\_ICCs}$ is one clock cycle to read data from the SRAM to the ICCs; $T_{ICCs\_to\_SRAM}$ is one clock cycle to write the output of the ICCs to the SRAM; $T_{SRAM\_to\_SDRAM}$ are two clock cycles to write $c(i_k,j_k)$ and $c(i'_k,j'_k)$ back to the DDR4 SDRAM. Consequently, $T_{N_{co}}$ is ten clock cycles, and substituting $T_{N_{co}}$ into Eq.(21), the throughput is
  23 $$\begin{aligned} Throughput=\frac{Q_{max} * K_{max}*M_{max}*N_{max}*f_{max}}{10N_{co}\lceil \frac{M_{max}*N_{max}}{N_{co}}\rceil } ~~\text {(Mbps)}, \end{aligned}$$
  where $f_{max}$ is the maximum frequency of MIE hardware, and it has been found that the critical path lies in the fixed-point multiplication of the chaotic map implementation (PCM Computing in Fig. 13) in this exemplar design; $Q_{max}$ is the maximum number of bits representing a pixel; and *K* is the number of images encrypted simultaneously. In this work, all hardware components in the design were optimized for the highest speed.
- The hardware efficiency: The hardware efficiency is computed as
  24 $$\begin{aligned} Efficiency=\frac{Throughput}{ALMs} ~~\text {(Mbps/ALM)}. \end{aligned}$$
  Tables 23 and 24 summarize hardware metrics and power consumption. The throughput is 998.2 Mbps measured with the value of the simulation parameters as $f_{max}=51.99$ MHz, $K_{max}=8$, $M_{max}=1024$, $N_{max}=1024$, $Q_{max}=24$, and $N_{co}=8$. These metrics indicate that the exemplar design requires modest resources on the FPGA chip, while it achieves high throughput, as given in Table 24. The low efficiency is obtained because there is hardware sacrification to optimize the speed with the use of pipeline technique. Note from Table 23 that the amount of 96.1% RAM blocks is required for the entire MIE system, which is composed of several hardware modules such as NiOS, DRAM Controller, etc., for performing an end-to-end application. In fact, BRAM utilization is only counted for MIE-IP solely at 0.516%. The amount of hardware utilization for MIE-IP is less than 10% of the resource of the Intel Arria 10 SoC FPGA chip. The power consumption has been measured using the software tools by the FPGA chip manufacturer after complete compilation, placement, and routing.
- The latency: Considering the hardware latency, the analysis is based on the configurations of the proposed structures shown in Fig. 1 and their flowcharts in Fig. 4. The latency is defined as the time duration between the input of the first pixel of an image and the output of the first ciphertext pixel. It should be noted that the Bit Interleaving, Bit Pre-Processing (BPP), and Diffusion blocks are implemented with combinational logic (without flip-flops); therefore, these blocks operate combinationally and do not consume any clock cycles. Only the Permutation block requires memory read and write operations for pixel data. The S-box is also implemented with combinational logic and functions as a codebook; however, its delay depends on how it is utilized in each structure. Specifically, the S-boxes in Structures 1 and 2 do not incur any delay, whereas the S-boxes in Structure 3 require time for reading and writing pixel values from and to memory during the substitution process. In Structure 3, after the permutation and diffusion processes are completed for all pixels of an image, the substitution process is performed sequentially by reading the pixel values from memory and writing them back after substitution. Therefore, the latency of the proposed structures can be analyzed as follows. For Structures 1 and 2: The pixels in image $I_k$ are processed sequentially. In the exemplar design, both pixels $p(i_k,j_k+1)$ and $c(i_k,j_k-1)$ can be read simultaneously because they are stored in the same word of the SRAM. Therefore, only one SRAM read cycle is required. As described above, each permutation requires one SRAM read and one SRAM write cycle, denoted as $T_{reading\_SRAM}$ and $T_{writing\_SRAM}$, respectively. The latency is calculated as the time interval from when the first pixel *p*(1, 1) enters the encryption core to when the first ciphertext pixel *c*(1, 1) is produced. However, since the permutation operates over the entire image-space range, the overall latency corresponds to the time needed to complete encryption of all pixels in the smallest image across $N_e$ encryption rounds. The size of the smallest image is used in this calculation because its ciphertext pixels are returned first. Therefore, the latency of Structures 1 and 2 for $N_e$ rounds of encryption is given by
  25 $$\begin{aligned} Latency_{1,2}=N_e*M_{min}*N_{min}*\left( 2*T_{reading\_SRAM}+T_{writing\_SRAM}\right) ~~~~~~~~(clock~cycles) \end{aligned}$$
  where $M_{min}$ and $N_{min}$ are the size of smallest image in terms of the numbers of pixels. For Structure 3: Encryption is divided into two stages. In the first stage, all pixels are completely permuted and diffused. In the second stage, the pixels are substituted using the S-box. The total latency is determined by the time required to complete the permutation and diffusion processes over $N_e$ rounds, plus the time required for $(N_e - 1)$ rounds of substitution. Therefore, the latency of Structure 3 is given by
  26 $$\begin{aligned} Latency_3=\left( 2*N_e-1\right) *M_{min}*N_{min}*\left( T_{reading\_SRAM}+T_{writing\_SRAM}\right) ~~~~~~~~(clock~cycles) \end{aligned}$$
  In FPGA, it only needs one clock cycle to read/write to SRAM, which means $T_{reading\_SRAM}=T_{writing\_SRAM}=1/f_{max}$. Equations (25) and (26) become
  27 $$\begin{aligned} Latency_{1,2}=\frac{3*N_e*M_{min}*M_{min}}{f_{max}}~~~(seconds) \end{aligned}$$
  and
  28 $$\begin{aligned} Latency_3=\frac{2*\left( 2*N_e-1\right) *M_{min}*N_{min}}{f_{max}}~~~(seconds) \end{aligned}$$
  In the exemplar hardware design with $N_e=5$, $M_{min}=N_{min}=256$ (see Table 1), the estimated latencies using Eq. (27) for Structures 1 and 2 and Eq. (28) for Structure 3 are $Latency_{1,2}\approx 18.908$ milliseconds and $Latency_{3}\approx 22.689$ milliseconds, respectively.
- Comparison to other works: Regardless of single- and multiple-image encryption, structure, or block and stream ciphers, Table 25 shows the results of throughput and efficiency between the exemplar design and other non-chaotic and chaos-based ciphers for reference. The referenced works are ordered by the implementation target and by years of publication. The results indicate that the proposed hardware achieves throughput comparable to that of recent ciphers, i.e., LSPT-SC^77^ and LED (128 bit key) in^78^. The disadvantage is that the efficiency of the exemplar design is significantly lower than the others, except for RC4. The examplar design has low efficiency because the entire system in this work is implemented on hardware, rather than a combination of hardware and software as in AES-128^79^. In addition, the throughput is also dependent on the level of security expected by means of number of encryption rounds. Therefore, these numbers of metrics in Table 25 are meant only for reference purposes.

**Table 23:** Resource utilization and power consumption of the MIE-IP and MIE system.

Metrics	MIE-IP	MIE system
ALMs	20,222/251,680 (8.03%)	55,599/251,680 (22.09%)
Registers	25,866/503,360 (5.14%)	85,787/503,360 (17.04%)
Block memory bits	1,152/43,642,880 (0.26%)	32,397,108/43,642,880 (74.23%)
RAM blocks	11/2,131 (0.516%)	2,048/2,131 (96.1%)
DSP blocks	140/1,687 (8.29%)	143/1,687 (8.47%)
Power consumption (W)	1.423	6.828

**Table 24:** Speed and efficiency of the MIE-IP.

Speed	Maximum frequency (MHz)	51.99
	Throughput (Mbps)	998.2
Efficiency (Mbps/ALMs)		0.018

**Table 25:** Comparison of hardware metrics between the proposed system and other reported non-chaos-based and chaos-based ciphers.

Cipher	Year	Implementation target	Throughput (Mbps)	Efficiency
LED (64-bit key)^78^	2020	ASIC	680.3	0.191
LED (128-bit key)^78^	2020	ASIC	1010.1	0.284
RC4^80^	2000	FPGA	135.52	0.013
A5/1^81^	2004	FPGA	90.85	
E0^81^	2004	FPGA	93.36	
Moustique^82^	2008	FPGA	369	
Chaos-based stream cipher^83^	2014	FPGA	3682.87	0.411
Chaos-ring^84^	2020	FPGA	464.688	-
LSPT-SC^77^	2020	FPGA	992.21	0.29
LSP-SC^77^	2020	FPGA	1037.27	0.32
LST-SC^77^	2020	FPGA	1179.07	1.12
AES-128 (FSM Architecture)^79^	2020	FPGA	3485	2.591
AES-128 (Loop Unrolled)^79^	2020	FPGA	4342	0.488
AES-256^85^	2021	FPGA	278	-
CLEFIA (three configurations)^86^	2021	FPGA	990/818/696	-
4D memristive hyperchaos cipher^87^	2024	FPGA	163.84	-
**This exemplar design**	**2024**	FPGA	**998.2**	**0.018**

## Discussion

The MIE structures have been proposed and verified through exemplar simulation and hardware design. Some discussion of structure, performance, security, and hardware improvements is given below.

In terms of structural configuration, the proposed structures are generic models, so that they can be scalable for as many images with different sizes and different pixel depths as possible. Here, the processes of reshaping and padding pixels are not required. In addition, any chaotic map can be employed in the proposed structures. Images of a cohort are encrypted separately and in parallel. Due to the separate encryption, it allows any ciphertext images from the cohort to be decrypted separately.

In terms of performance, the simulation shows that all images have the same number of encryption rounds, $N_e$. So, encryption for small images will be completed earlier than for larger ones. In fact, the proposed structures can encrypt different images with different numbers of encryption rounds by setting the condition for flags $Pause_k$. Unlike most existing algorithms of chaos-based MIE, the permutation and diffusion are combined in the same step, so it saves the time of chaotic iterations in generation of session keys.

In terms of security, with the inclusion of an S-box and session keys of image content dependency, the ciphers employing the proposed structures are sensitive to the image content and can resist any type of attack that exploits the difference in the plaintexts and ciphertexts. In addition, the key space can be expanded by lengthening the secret key in several ways, i.e., increasing the number of bits representing chaotic values, choosing a higher-dimensional chaotic map, and lengthening the size of the LFSR. However, these solutions require increasing computational complexity.

In terms of hardware performance, the hardware design for the example using Structure 1 has been synthesized and implemented on the FPGA platform, in which the value of parameters is chosen as given in the simulation. The timing of the hardware design is optimized for the speed of the MIE core. That is, the pipeline technique is used for the design of PCM, SKS, and MRWCs. The speed of the hardware is improved because the SRAM in the FPGA chip is used to temporarily store pixels for encryption. According to Eq. (23), for a certain maximum frequency of the MIE-IP core, $f_{max}$, the throughput can be scalable by the number of images to be encrypted at the same time, $K_{max}$, as well as by the number of bits representing pixels, $Q_{max}$. In fact, the trade-off between the throughput and the utilization of hardware resource must be considered in this case. It is noted that the other hardware designs listed in Table 25 cannot be scaled to have the desired throughput value. In addition, the hardware design based on Structures 2 and 3 is almost the same as that given in the exemplar implementation of Structure 1, except for the position of the S-box relative to the diffusion. The S-box is implemented as a codebook by logic gates, so it does not take any clock cycle.

## Conclusion

In summary, three chaos-based MIE models have been proposed that allow encrypting multiple images in parallel, and the images of the cohort can be various in sizes and pixel depths. Any selected individual ciphertext images from the cohort can be decrypted separately. The simulation results and hardware implementation for the example show the effectiveness and feasibility of the proposed structures. The proposed models are suitable for the application of diverse image data.

In these models, the encryption is performed pixel by pixel sequentially for an entire image, rather than blocks of pixels, therefore, it is sensitive to any change in the value of ciphertext pixels. This means that there are challenges for the types of attack based on the analysis of pairs of plaintexts and ciphertexts. However, the proposed structures are not robust to the ciphertext-crop attack. In future work, the proposed structures will be further developed to improve both the speed and efficiency of the hardware, and the security to resist the ciphertext-crop attack.

## Data Availability

The datasets used and/or analysed during the current study available from the corresponding author on reasonable request.

## Code availability

The code used for simulation, design, and analysis during the current study is available from the corresponding author on reasonable request.
